# *Salmonella* Enteritidis T1SS protein SiiD inhibits NLRP3 inflammasome activation via repressing the mtROS-ASC dependent pathway

**DOI:** 10.1371/journal.ppat.1011381

**Published:** 2023-05-08

**Authors:** Yaxin Guo, Dan Gu, Tingting Huang, Ang Li, Yi Zhou, Xilong Kang, Chuang Meng, Dan Xiong, Li Song, Xinan Jiao, Zhiming Pan

**Affiliations:** 1 Jiangsu Key Laboratory of Zoonosis, Yangzhou University, Yangzhou, Jiangsu, China; 2 Jiangsu Co-innovation Center for Prevention and Control of Important Animal Infectious Diseases and Zoonoses, Yangzhou University, Jiangsu, China; 3 Key Laboratory of Prevention and Control of Biological Hazard Factors (Animal Origin) for Agrifood Safety and Quality, Ministry of A griculture of China, Yangzhou University, Yangzhou, Jiangsu, China; 4 Joint International Research Laboratory of Agriculture and Agri-product Safety of the Ministry of Education, Yangzhou University, Jiangsu, China; 5 School of Nursing School of Public Health, Yangzhou University, Jiangsu, China; University of Illinois, UNITED STATES

## Abstract

Inflammasome activation is an essential innate immune defense mechanism against *Salmonella* infections. *Salmonella* has developed multiple strategies to avoid or delay inflammasome activation, which may be required for long-term bacterial persistence. However, the mechanisms by which *Salmonella* evades host immune defenses are still not well understood. In this study, *Salmonella* Enteritidis (SE) random insertion transposon library was screened to identify the key factors that affect the inflammasome activation. The type I secretion system (T1SS) protein SiiD was demonstrated to repress the NLRP3 inflammasome activation during SE infection and was the first to reveal the antagonistic role of T1SS in the inflammasome pathway. SiiD was translocated into host cells and localized in the membrane fraction in a T1SS-dependent and partially T3SS-1-dependent way during SE infection. Subsequently, SiiD was demonstrated to significantly suppress the generation of mitochondrial reactive oxygen species (mtROS), thus repressing ASC oligomerization to form pyroptosomes, and impairing the NLRP3 dependent Caspase-1 activation and IL-1β secretion. Importantly, SiiD-deficient SE induced stronger gut inflammation in mice and displayed NLRP3-dependent attenuation of the virulence. SiiD-mediated inhibition of NLRP3 inflammasome activation significantly contributed to SE colonization in the infected mice. This study links bacterial T1SS regulation of mtROS-ASC signaling to NLRP3 inflammasome activation and reveals the essential role of T1SS in evading host immune responses.

## Author summary

*Salmonella* Enteritidis (SE) is a Gram-negative, non-host specific, facultative intracellular pathogen. SE infection is one of the most important global public health security problems. In recent years, SE has become the predominant serotype of *Salmonella* responsible for salmonellosis. Inflammasome activation plays an important role in the host immune defense against pathogen infections. However, the knowledge of the interactions between SE and inflammasomes is limited. How SE modulates inflammasome activation to evade host immune clearance and achieve persistent infection remains poorly understood. Our study demonstrates that bacterial T1SS protein SiiD is involved in the inhibition of NLRP3 inflammasome activation during SE infection. More importantly, we revealed the molecular mechanism by which SiiD depresses NLRP3 inflammasome signaling and subsequently promotes the virulence and colonization of SE *in vivo*. Our findings expand the understanding of the contribution of *Salmonella* T1SS in counteracting host immune responses mediated by the NLRP3 inflammasome.

## Introduction

Inflammasomes are macromolecular multimeric protein complexes that are assembled by intracellular pattern recognition receptors (PRRs), such as Nod-like receptors (NLRs), the adaptor protein apoptosis-associated speck-like protein containing a CARD (ASC), and the effector pro-Caspase-1 [1]. Inflammasomes can recognize conserved pathogen-associated molecular patterns (PAMPs), microbial-associated molecular patterns (MAMPs), and host-derived danger-associated molecular patterns (DAMPs) [2]. Inflammasome activation initiates self-cleavage and Caspase-1 activation. Active Caspase-1 then proteolytically matures the pro-inflammatory cytokines pro-IL-1β and pro-IL-18 into their biologically active, secreted forms [3]. The N-terminal cleavage of the pore-forming protein gasdermin D (GSDMD), mediated by Caspase-1/4/5/11, leads to the formation of plasma membrane GSDMD pores and a specialized form of pro-inflammatory programmed necrosis termed pyroptosis [4]. Activation of the inflammasomes is an essential mechanism of the innate immune response against pathogenic microbial infection. The secretion of pro-inflammatory cytokines IL-1β and IL-18, release of inflammatory cytoplasmic contents, and further activation of other innate immune effectors mediated by pyroptosis are important for the clearance of pathogenic microbes *in vivo* [5,6].

*Salmonella enterica* subsp. *enterica* serotype Enteritidis (*Salmonella* Enteritidis, SE) is one of the major foodborne zoonotic pathogens worldwide. There are approximately 90 million cases of diarrhea-associated diseases caused by *Salmonella* worldwide annually, with 85% of these cases linked to food [7]. The expected global fatality rate associated with salmonellosis is over 200,000 [8]. Fatalities are most often observed in children below the age of 4 years who are infected with SE or *Salmonella* Typhimurium (ST) [9]. SE is most often responsible for food poisoning, a recent case reported that SE can also cause brain abscess [10]. In essence, the ability to invade, survive and replicate within the host phagocytes are crucial for *Salmonella* to establish systemic infection in hosts. *Salmonella* pathogenicity island-1 (SPI-1)-encoded type III secretion system-1 (T3SS-1) is essential for *Salmonella* to invade into intestinal epithelial cells [11], and bacteria subsequently replicate within a specialized *Salmonella*-containing vacuole (SCV), which is established and maintained by SPI-2-encoded T3SS-2 [12]. More than 60 effectors can be secreted and translocated into the host cells via T3SS to regulate host cell progression and evade host immune responses to establish persistent infection [13].

The essential role of inflammasome activation in mediating pyroptosis and pro-inflammatory cytokines secretion in the immune response against *Salmonella* infection has been gradually appreciated. Mice lacking both NLRC4 and NLRP3 are significantly more susceptible to *Salmonella* infection [14]. In addition, the *Casp1*^-/—^, *IL-1β*^-/—^, and *IL-18*^-/—^mice also succumbed to *Salmonella* infection more rapidly than wild-type (WT)-C57BL/6 mice, with markedly higher bacterial burdens in spleens, Peyer’s patches, and mesenteric lymph nodes [15]. Flagellin, T3SS rod, and needle protein injected into host cells by invading *Salmonella*, can be sensed by NLRC4 and NLRP3 inflammasome [16]. Therefore, the expression of SPI-1 and flagellin is strongly inhibited in the intracellular environment by activating SPI-2 [17]. This evasion strategy appears to be efficient but not perfect. NLRC4 inflammasome can still detect *Salmonella* with suppressed flagellin/SPI-1 expression at late times post-infection [14]. To survive and replicate within the host cells, *Salmonella* has evolved multiple mechanisms to avoid or delay inflammasome activation, many of which remain largely unknown. Therefore, further exploration of the molecular mechanisms by which SE evades inflammasome activation is essential for elucidating *Salmonella* pathogenicity mechanisms, as well as for developing new therapeutic strategies against SE infection.

Flagellin is highly induced in the late logarithmic growth phase of SE and induce robust inflammasome activation within 1 h *in vitro* [17]. To exclude any possible interference by flagellin, a transposon mutant library based on the SE flagellin-deficient strain C50336Δ*fliC* (Δ*fliC*) was constructed and screened for potential genes involved in regulating inflammasome activation. We found that *siiD*, the gene encoding the T1SS HlyD family protein, was essential for SE to repress the activation of NLRP3 inflammasome. Subsequent studies revealed that SE T1SS protein SiiD suppressed the generation of mtROS, which is required for the formation of ASC pyroptosome, and thus inhibited the activation of NLRP3 inflammasome and secretion of pro-inflammatory cytokines. Furthermore, SiiD-deficient SE exhibited defects in virulence and colonization capacity after oral administration. This study identified the first T1SS gene of *Salmonella* that mediate the evasion of NLRP3 inflammasome-mediated host immune clearance, which provides new insights into the prevention and control measures against SE infection.

## Results

### Screening and identification of potential genes modulated the inflammasome activation

Since *Salmonella* flagellin can induce rapid and strong inflammasome activation, a *mariner*-based transposon (TnpSC189) mutant library in a flagellin-deficient strain of SE (Δ*fliC*) was generated and screened to identify other potentially critical genes involved in modulating inflammasome activation.

**Fig 1:**
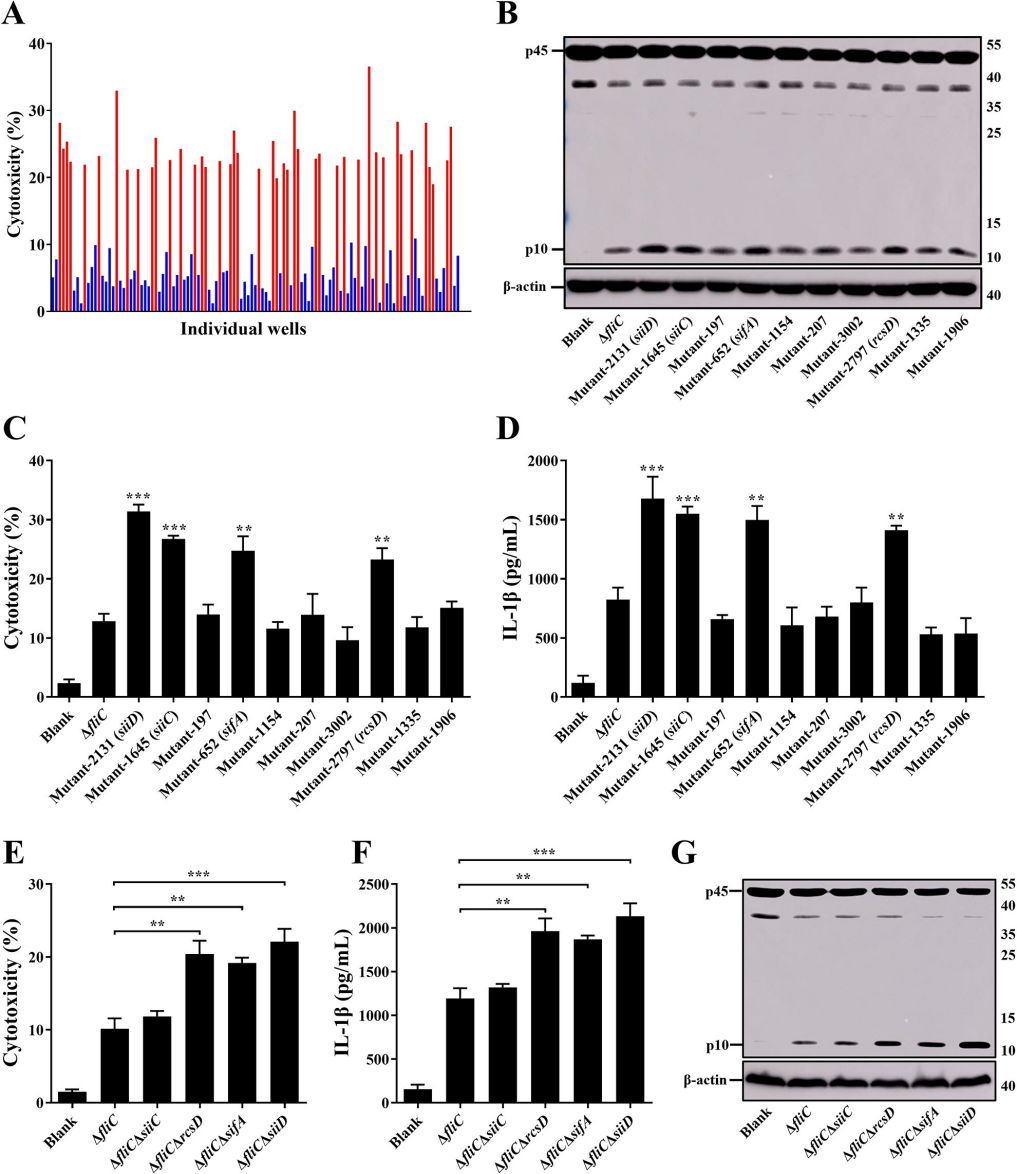
Screening Δ*fliC* transposon mutant library for identification of the key factors involved in modulating inflammasome activation *in vitro*. J774A.1 cells were primed with LPS (1 μg/mL, 5 h) and then infected with Δ*fliC* transposon mutants or gene deletion mutants at an MOI of 100 for 4.5 h, uninfected cells were used as a negative control (Blank). (**A**, **C**, **E**) Culture supernatants were analysed for cytotoxicity evaluated by LDH release. (**A**) The Z score was calculated for each well in a 48-well cell plate, and a Z score ≤ -2 or ≥ 2 was considered significant. 43 mutants induced significantly higher cytotoxicity levels (red, Z score ≥ 2) and 72 mutants induced significantly lower cytotoxicity levels (blue, Z score ≤ -2). (**D**, **F**) IL-1β secretion in supernatants were examined via ELISA. (**B**, **G**) The activation of Caspase-1 (p10) was analyzed by western blotting. β-actin was blotted as a loading control. Molecular mass markers in kDa are indicated on the right. Data are presented as mean ± SEM of triplicate samples per experimental condition from three independent experiments. **p < 0.01, ***p < 0.001, as measured by one-way ANOVA followed by Bonferroni’s multiple comparison test.

The candidate mutants were selected by increased or decreased Lactate dehydrogenase (LDH) release relative to the Δ*fliC* parental strain, 4.5 h after infection of the mouse macrophage cell line J774A.1. So far, 3409 transposon mutants have been individually verified. Following the first-round of screening, 115 mutants were considered as candidates for the second-round of validation (Fig 1A). 72 mutants (Z score ≤ -2) induced significantly lower cytotoxicity, whereas 43 mutants (Z score ≥ 2) induced significantly higher cytotoxicity than that of Δ*fliC*. In the second screening round, each candidate mutant was tested individually in J774A.1 cells. LDH release was quantified and Caspase-1 activation was determined by western blotting. The candidate transposon mutants 2131, 1645, 652, and 2797 induced significantly higher levels of cytotoxicity and Caspase-1 activation compared to Δ*fliC* (Fig 1B and 1C). Transposon insertion sequencing of the candidate mutants identified these four genes as *siiD*, *siiC*, *sifA*, and *rcsD*; the transposon insertion sites were shown in S1B Fig. The *siiD* encodes a T1SS HlyD family protein, *siiC* encodes a T1SS TolC family protein, *sifA* encodes a SPI-2 T3SS effector, and *rcsD* encodes a phosphotransferase.

The in-frame deletion mutant strains of *siiD*, *siiC*, *sifA*, and *rcsD* were constructed using the suicide plasmid pDM4 to further investigate the function of these genes in regulating inflammasome activation. As shown in Fig 1E, infection with Δ*fliC*Δ*siiD*, Δ*fliC*Δ*sifA*, and Δ*fliC*Δ*rcsD* resulted in greatly increased LDH release in comparison with J774A.1 cells infected by Δ*fliC*. We subsequently examined whether the increased cell death was caused by inflammasome activation. As expected, the production of cleaved Caspase-1 p10 subunits and Caspase-1-dependent cytokine IL-1β was significantly increased in the cells infected with Δ*fliC*Δ*siiD*, Δ*fliC*Δ*sifA*, or Δ*fliC*Δ*rcsD* compared to Δ*fliC* (Fig 1F and 1G). These results suggested that SiiD, SifA, and RcsD inhibited the activation of inflammasome during SE infection. The *siiD*, which showed the strongest ability to inhibit inflammasome activation, was selected as the target gene for subsequent research.

### SiiD specifically inhibited the activation of NLRP3 inflammasome

To further confirm the role of SiiD in repressing inflammasome activation, the complemented strain Δ*fliC*Δ*siiD*::*siiD* and the empty vector complemented strain Δ*fliC*Δ*siiD*::Vector were constructed using the plasmid pBAD33. There was no significant difference in growth characteristics and invasiveness among the parental, mutant, complemented, and empty vector-complemented strains (S1C and S1D Fig). Furthermore, the transcript levels of *siiABCDEF* and T3SS-1 apparatus (*prgHIJK*, *spaPQRS*, *sipBCD*, and *invAG*) genes were no significant difference in the indicated SE after infecting the J774A.1 cells (S1E and S1F Fig). These results indicated that the deficiency of SiiD did not affect the normal expression of T1SS and T3SS-1.

**Fig 2:**
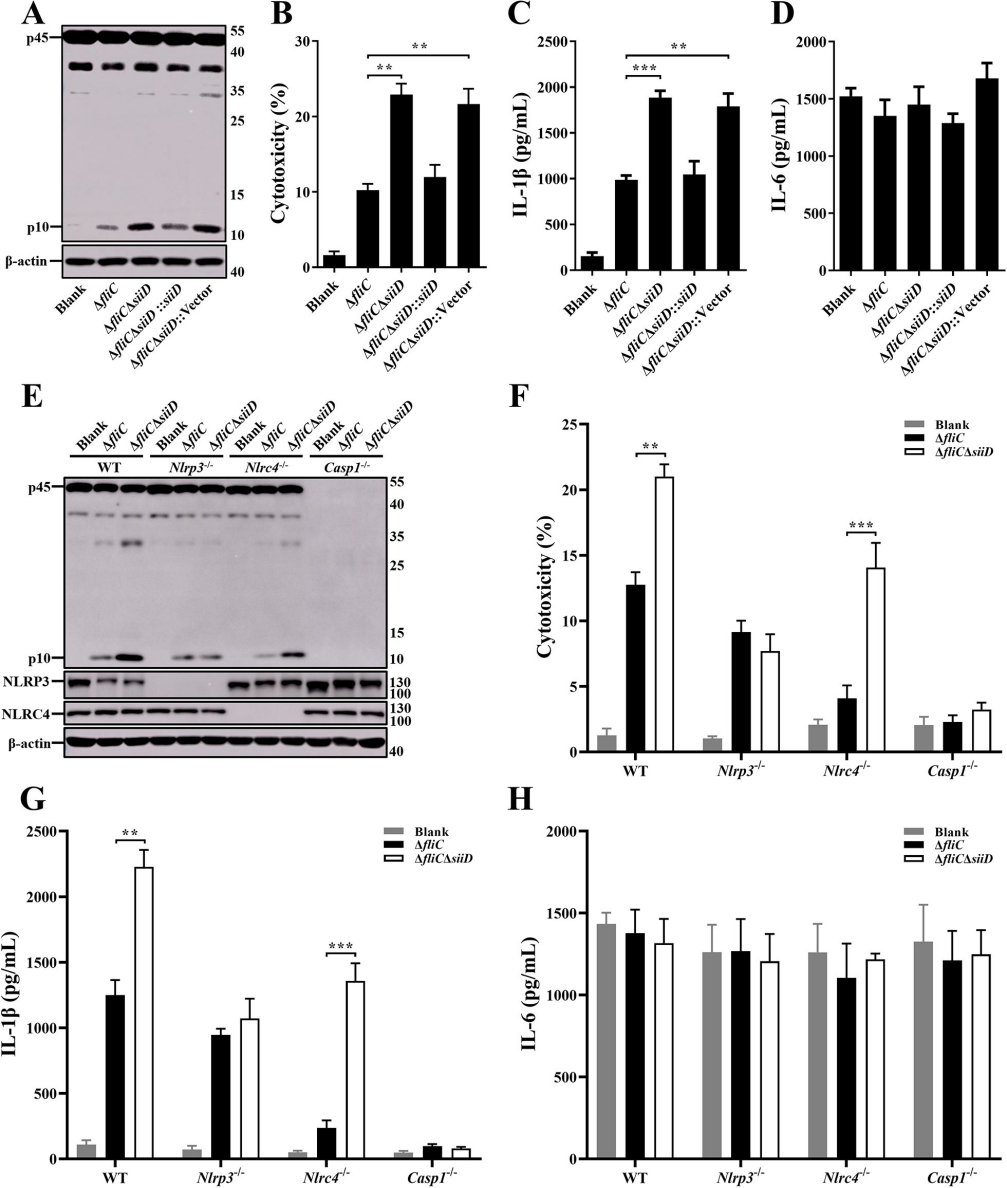
SiiD specifically repressed NLRP3 inflammasome activation. BMDMs from WT-, *Nlrp3*^-/—^, *Nlrc4*^-/—^, and *Casp1*^-/—^C57BL/6 mice were primed with LPS (200 ng/mL, 5 h). The *Nlrc4*^-/—^BMDMs were infected with Δ*fliC*, Δ*fliC*Δ*siiD*, Δ*fliC*Δ*siiD*::*siiD*, or Δ*fliC*Δ*siiD*::Vector at an MOI of 100:1 for 4.5 h. The WT-, *Nlrp3*^-/—^, and *Casp1*^-/—^BMDMs were infected with indicated bacteria at an MOI of 50:1 for 4.5 h, uninfected cells were used as a negative control (Blank). (**A**, **E**) The activation of Caspase-1 (p10) and the expression of NLRP3 and NLRC4 were analyzed by western blotting. β-actin was blotted as a loading control. Molecular mass markers in kDa are indicated on the right. (**B**, **F**) Culture supernatants were analysed for cytotoxicity evaluated by LDH release. (**C**, **G**) IL-1β and (**D**, **H**) IL-6 secretion in supernatants were examined via ELISA. Data are presented as mean ± SEM of triplicate samples per experimental condition from three independent experiments. **p < 0.01, ***p < 0.001, as measured by one-way ANOVA followed by Bonferroni’s multiple comparison test.

Primary bone marrow-derived macrophages (BMDMs) obtained from WT-C57BL/6 mice were infected with the indicated SE strains. The cytotoxicity of BMDMs induced by Δ*fliC*Δ*siiD* or Δ*fliC*Δ*siiD*::Vector was significantly enhanced compared to Δ*fliC*, as well as Caspase-1 activation and IL-1β secretion (Fig 2A–2C). The ability of inducing inflammasome activation was recovered to a normal level when infected with Δ*fliC*Δ*siiD*::*siiD*. Simultaneously, to further verify whether SiiD can specifically influence the inflammasome pathway, we also measured the levels of the non-inflammasome inflammatory cytokine IL-6 in the supernatants. However, there was no significant difference in IL-6 secretion among the indicated SE strains-infected cells (Fig 2D), suggesting that *siiD* specifically repressed the activation of inflammasome.

The BMDMs obtained from WT-, *Nlrp3*^-/—^, *Nlrc4*^-/—^, and *Casp1*^-/—^C57BL/6 mice were infected with the indicated SE strains to confirm the specific type of inflammasome inhibition by SiiD. The Δ*fliC*Δ*siiD* significantly induced Caspase-1 activation, cell death, and IL-1β secretion in WT- and *Nlrc4*^-/—^BMDMs, but not in *Nlrp3*^-/—^BMDMs (Fig 2E–2G). The signals of inflammasome activation were not detected in *Casp1*^-/—^BMDMs. The expression of IL-6 was not affected by SiiD in any of the BMDMs (Fig 2H). Taken together, these results demonstrated that SE T1SS SiiD protein specifically inhibited the NLRP3 inflammasome activation.

### Identification of SE T1SS protein SiiD translocated into host cells

**Fig 3:**
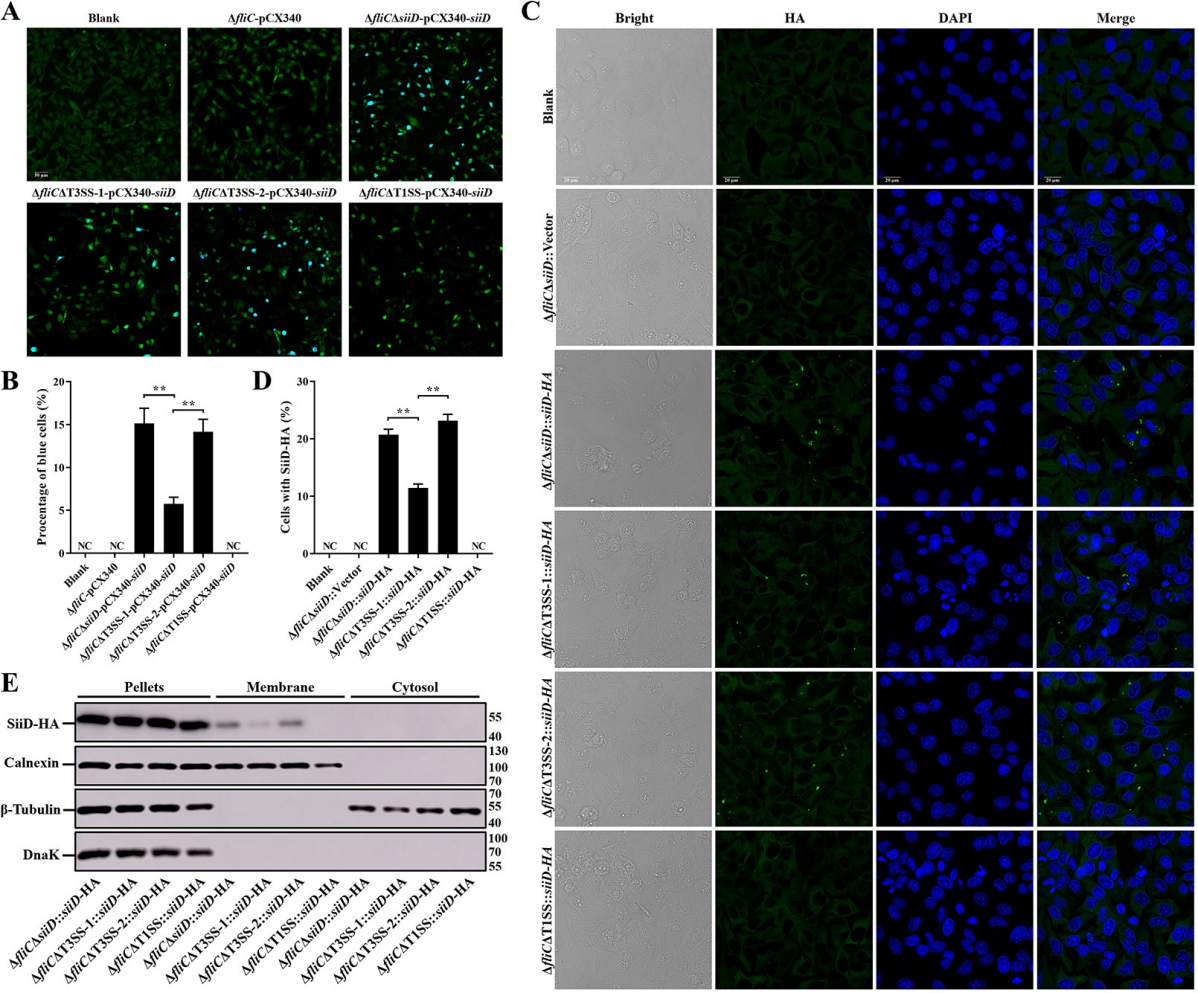
The T1SS-dependent translocation and membrane localization of SiiD. HeLa cells were infected with Δ*fliC*, Δ*fliC*ΔT3SS-1, Δ*fliC*ΔT3SS-2, or Δ*fliC*ΔT1SS bearing empty plasmid pCX340 or expressing SiiD-TEM-1 fusion protein at an MOI of 100 for 8 h, uninfected HeLa cells was used as a negative control (Blank). (**A**) Cells were then loaded with CCF2-AM. Translocation of SiiD-TEM-1 fusion protein into the cell cytosol results in cleavage of CCF2-AM, emission of blue fluorescence, whereas uncleaved CCF2-AM emitted green fluorescence. Scale bar, 50 μm. (**B**) The percentages of cells emitting blue fluorescence. Approximately 600 cells were counted in each sample. HeLa cells were infected with Δ*fliC*Δ*siiD*, Δ*fliC*ΔT3SS-1, Δ*fliC*ΔT3SS-2, or Δ*fliC*ΔT1SS bearing empty plasmid pBAD33 or expressing SiiD-HA fusion protein at an MOI of 100 for 8 h, uninfected HeLa cells was used as a negative control (Blank). (**C**) The intracellular translocation of SiiD-HA was detected by indirect immunofluorescence assay. SiiD-HA, green; DAPI, blue. Scale bar, 20 μm. (**D**) The percentage of cells with SiiD-HA green fluorescent spots. Approximately 200 cells were counted in each sample. (**E**) HeLa cells were resuspended and lysed after infection. HeLa cells were subjected to differential centrifugation to separate subcellular fractions. The cytosol fraction, membrane fraction, and pellets fraction were analyzed by western blotting. Calnexin and β-tubulin, markers of HeLa cells membrane and cytosolic proteins, respectively. DnaK, marker of bacterial protein. Data are presented as mean ± SEM of triplicate samples per experimental condition from three independent experiments. **p < 0.01, as measured by one-way ANOVA followed by Bonferroni’s multiple comparison test.

*Salmonella* secretion systems, essential for bacterial pathogenicity, could transfer the effectors into host cells to interfere cellular processes, evade host immune defense, and establish persistence infection. T3SS is one of the most important secretion systems of *Salmonella*, whereas T1SS is necessary for *Salmonella* invasion into polarized epithelial cells [18]. The fluorescence resonance energy transfer (FRET) assay was used to examine whether the SE T1SS protein SiiD can be translocated into host cells by T3SS or T1SS. As shown in Fig 3A, uninfected HeLa cells and cells infected with Δ*fliC*-pCX340 emitted green fluorescence after loading them with CCF2-AM, suggesting the absence of TEM-1 activity in these cells. Approximately 15.1% of HeLa cells infected with Δ*fliC*Δ*siiD* strain expressing SiiD-TEM-1 fusion protein emitted blue fluorescence, whereas the number of blue cells was significantly decreased upon infection with Δ*fliC*ΔT3SS-1-pCX340-*siiD*. However, T3SS-2 deficiency did not affect the translocation of SiiD-TEM-1. However, no blue fluorescence was detected in the HeLa cells infected with Δ*fliC*ΔT1SS-pCX340-*siiD*.

In addition, the translocation of SiiD-HA was directly observed by indirect immunofluorescence assay. Similarly, green fluorescent spots were observed in HeLa cells infected with Δ*fliC*Δ*siiD*::*siiD*-HA or Δ*fliC*ΔT3SS-2::*siiD*-HA (Fig 3C and 3D). Deletion of T3SS-1 significantly reduced the number of cells with green fluorescent spots, whereas no green fluorescent spots were observed in uninfected cells and cells infected with Δ*fliC*Δ*siiD*::Vector or Δ*fliC*ΔT1SS::*siiD*-HA. Furthermore, our results also showed that the SiiD protein was localized in the membrane fraction after its translocation into HeLa cells (Fig 3E). The amount of SiiD-HA protein localized in the membrane fraction from Δ*fliC*ΔT3SS-1::*siiD*-HA infected cells was significantly decreased compared to Δ*fliC*Δ*siiD*::*siiD*-HA or Δ*fliC*ΔT3SS-2::*siiD*-HA infected cells. In contrast, no SiiD-HA protein was detected in the membrane fraction of cells infected with Δ*fliC*ΔT1SS::*siiD*-HA. These data indicated that SiiD may be a T1SS effector of SE, which was translocated into host cells and localized in the membrane fraction in a T1SS-dependent, partially T3SS-1-dependent, and T3SS-2-independent ways.

### SiiD independently inhibited NLRP3 inflammasome activation

**Fig 4:**
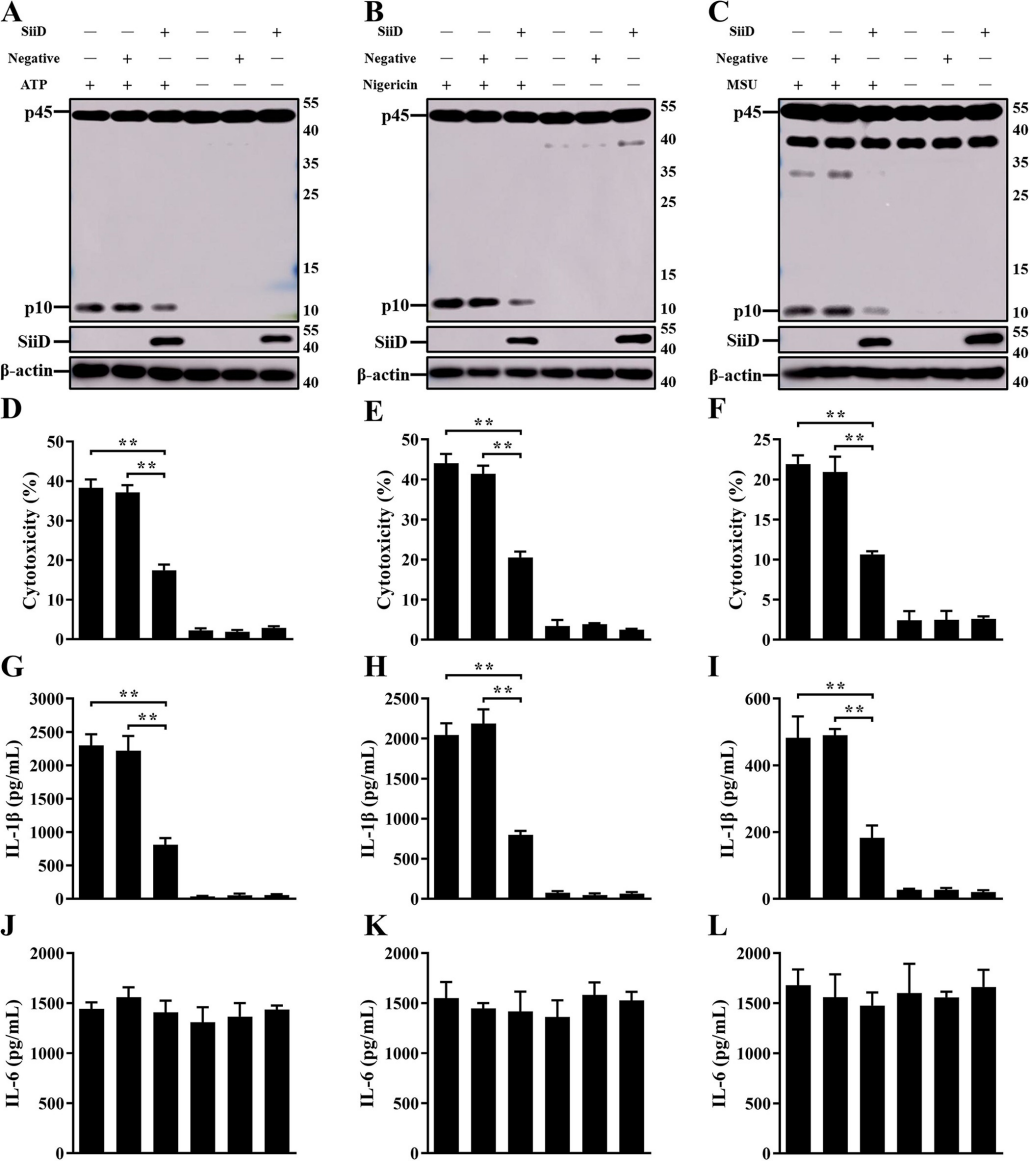
SiiD independently suppressed NLRP3 inflammasome activation. J774A.1 cells were transduced with Lv-SiiD or Lv-NC lentivirus, untreated cells were used as a negative control (Blank). Cells were then primed with LPS (1 μg/mL, 5 h) and stimulated with or without ATP (1.25 mM) or nigericin (10 μM) for 1 h or MSU crystals (200 μg/mL) for 6 h. (**A**, **B**, **C**) The activation of Caspase-1 (p10) and the expression of SiiD were analyzed by western blotting. β-actin was blotted as a loading control. Molecular mass markers in kDa are indicated on the right. (**D**, **E**, **F**) Supernatants were analysed for cytotoxicity evaluated by LDH release. (**G**, **H**, **I**) IL-1β and (**J**, **K**, **L**) IL-6 secretion in supernatants were examined via ELISA. Data are presented as mean ± SEM of triplicate samples per experimental condition from three independent experiments. **p < 0.01, as measured by one-way ANOVA followed by Bonferroni’s multiple comparison test.

To further verify the inhibitory effect of SiiD on NLRP3 inflammasome activation, the lentiviral vector pGLV5-*siiD* (EF-1aF/GFP&Puro) was constructed to heterologously express the SiiD protein in J774A.1 cells. SiiD protein was detected in cells transfected with the LV5-SiiD lentivirus (Fig 4A–4C), indicating that the SiiD protein can be expressed in the macrophages infected with lentivirus. NLRP3 inflammasome activators like ATP, nigericin, and MSU crystals were employed to replace SE infection. After stimulation, cytotoxicity, IL-1β secretion, and Caspase-1 activation were significantly decreased in SiiD-expressing J774A.1 cells compared to those in control cells and negative lentivirus-transfected cells (Fig 4A–4I). However, the expression of IL-6 was not suppressed by SiiD (Fig 4J–4L). These results suggested that the SE T1SS protein SiiD could independently inhibit NLRP3 inflammasome activation in mouse macrophages.

### SiiD inhibited the activation of NLRP3 inflammasome by suppressing mtROS generation

To further explain the molecular mechanism of SiiD-mediated inhibition of NLRP3 inflammasome activation, we investigated whether SiiD was involved in regulating the expression of various component proteins of the NLRP3 inflammasome. The expression levels of NLRP3, ASC, pro-Caspase-1, and pro-IL-1β were not significantly different in LPS pretreated and non-pretreated macrophages infected with the indicated SE strains (S2A and S2B Fig), suggesting that SiiD repressed NLRP3 inflammasome activation was not through influencing the expression of NLRP3 inflammasome component proteins. NLRP3 inflammasome functions as a signal integrator that senses several cellular signals, including K^+^ efflux, Ca^2+^ influx, lysosomal rupture, and reactive oxygen species (ROS) production [19]. Next, we examined whether SiiD was involved in regulating these signals. There was no significant difference in the intracellular K^+^ and Ca^2+^ fluxes induced by Δ*fliC* and Δ*fliC*Δ*siiD* (S2C–S2F Fig). Cells treated with the lysosomal rupture inducer Leu-Leu-OMe/HCl (LLOMe, Santa Cruz Biotechnology, Dallas, TX, USA) were used as a positive control to test whether SiiD repressed lysosomal rupture in infected cells. However, no significant difference was found in the intensity of red fluorescence from macrophages infected with Δ*fliC* and Δ*fliC*Δ*siiD*, whereas red fluorescence emitted from intact lysosomes was undetectable in LLOMe-treated cells (S2G and S2H Fig). These results demonstrated that SiiD-mediated inhibition of NLRP3 inflammasome activation was not through repressing the K^+^/Ca^2+^ fluxes and lysosomal rupture.

**Fig 5:**
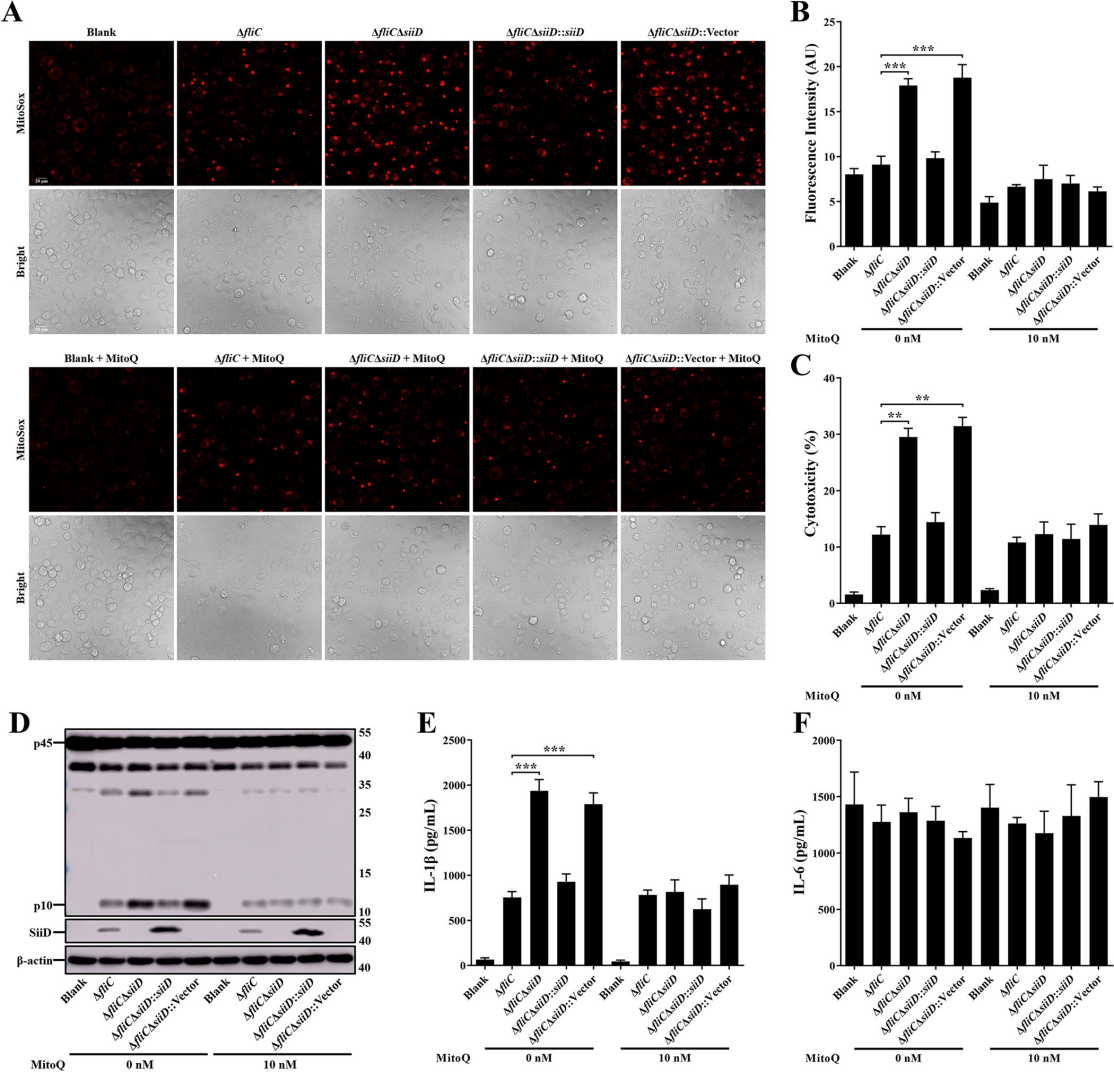
SiiD inhibited NLRP3 inflammasome activation by inhibiting mtROS generation. WT-BMDMs were primed with LPS (200 ng/mL) for 5 h, MitoQ (10 nM) or vehicle control (DMSO) was added to cells 1 h after LPS treatment. Then cells were infected with Δ*fliC*, Δ*fliC*Δ*siiD*, Δ*fliC*Δ*siiD*::*siiD*, or Δ*fliC*Δ*siiD*::Vector at an MOI of 50:1 for 4.5 h, uninfected cells were used as a negative control (Blank). (**A**) Cells were then loaded with MitoSOX Red (5 μM) for 30 min. Production of mitochondrial superoxide in infected cells was assayed. Scale bar, 20 μm. (**B**) The mean MitoSOX red fluorescence was quantified using Application Suite software. (**C**) Supernatants were analysed for cytotoxicity evaluated by LDH release. (**D**) The activation of Caspase-1 (p10) and the expression of SiiD were analyzed by western blotting. β-actin was blotted as a loading control. Molecular mass markers in kDa are indicated on the right. (**E**) IL-1β and (**F**) IL-6 secretion in supernatants were examined via ELISA. Data are presented as mean ± SEM of triplicate samples per experimental condition from three independent experiments. **p < 0.01, ***p < 0.001, as measured by one-way ANOVA followed by Bonferroni’s multiple comparison test.

The intracellular mtROS generation was detected using the MitoSOX Red mitochondrial superoxide indicator. The results showed that the Δ*fliC*Δ*siiD* and Δ*fliC*Δ*siiD*::Vector induced a significant enhancement of mtROS compared to Δ*fliC* or Δ*fliC*Δ*siiD*::*siiD* infected BMDMs (Fig 5A and 5B), suggesting that SiiD might inhibit the NLRP3 inflammasome activation by depressing intracellular mtROS generation. To further verify this conjecture, intracellular mtROS was scavenged using the mitochondria-targeted antioxidant MitoQ before SE infection. The results showed that MitoQ significantly counteracted the enhancement of mtROS generation induced by Δ*fliC*Δ*siiD* or Δ*fliC*Δ*siiD*::Vector (Fig 5A and 5B). In addition, pretreatment of BMDMs with MitoQ also significantly inhibited cell death, Caspase-1 activation, and IL-1β secretion in case of Δ*fliC*Δ*siiD* or Δ*fliC*Δ*siiD*::Vector infection (Fig 5C–5E). Furthermore, similar results were observed in MitoQ-treated J774A.1 cells infected with the indicated SE strains. We found that Δ*fliC*Δ*siiD* or Δ*fliC*Δ*siiD*::Vector induced significantly higher level of mtROS and inflammasome activation compared to Δ*fliC* or Δ*fliC*Δ*siiD*::*siiD* in untreated J774A.1 cells (S3 Fig). Whereas, the capacity of Δ*fliC*Δ*siiD* and Δ*fliC*Δ*siiD*::Vector to induce mtROS and inflammasome activation was restored to a normal level in MitoQ-treated J774A.1 cells. These results confirmed that SiiD suppressed the activation of NLRP3 inflammasome by preventing intracellular mtROS generation.

### SiiD suppressed the formation of NLRP3-dependent ASC pyroptosome

**Fig 6:**
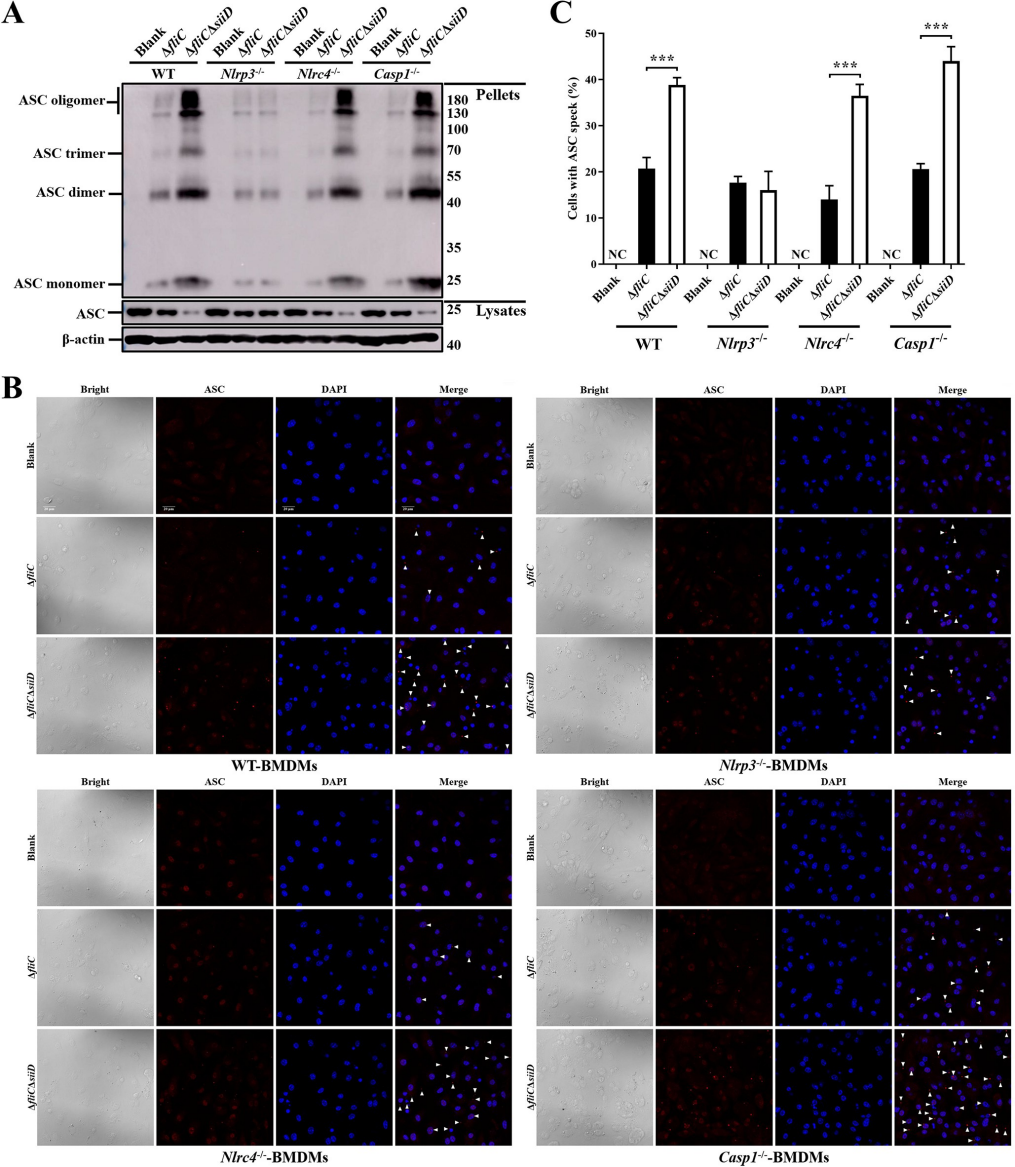
SiiD inhibited the formation of NLRP3 dependent ASC pyroptosome. BMDMs from WT-, *Nlrp3*^-/—^, *Nlrc4*^-/—^, and *Casp1*^-/—^C57BL/6 mice were primed with LPS (200 ng/mL, 5 h). The *Nlrc4*^-/—^BMDMs were infected with Δ*fliC* or Δ*fliC*Δ*siiD* at an MOI of 100:1 for 4.5 h. The WT-, *Nlrp3*^-/—^, and *Casp1*^-/—^BMDMs were infected with indicated bacteria at an MOI of 50:1 for 4.5 h, uninfected cells were used as a negative control (Blank). (**A**) Cells were lysed after infection and the pellets were subjected into cross-linking. The ASC oligomerization in the pellets and the total ASC in lysates as the input were examined by western blotting. β-actin was blotted as a loading control. Molecular mass markers in kDa are indicated on the right. (**B**) The formation of ASC specks (arrowheads) in infected BMDMs were detected by indirect immunofluorescence assay. ASC, red; DAPI, blue. Scale bar, 20 μm. (**C**) The percentages of cells with ASC speck. Approximately 200 cells were counted in each sample. Data are presented as mean ± SEM of triplicate samples per experimental condition from three independent experiments. ***p < 0.001, as measured by unpaired *t* test.

Formation of the ASC pyroptosome is essential for NLRP3 inflammasome activation. Subsequently, the involvement of SiiD in the regulation of ASC pyroptosome formation was examined. WT-, *Nlrp3*^-/—^, *Nlrc4*^-/—^, and *Casp1*^-/—^BMDMs were obtained and infected with the indicated SE strains. The formation of ASC dimers, trimers, and oligomers was significantly enhanced in WT-, *Nlrc4*^-/—^, and *Casp1*^-/—^BMDMs infected with Δ*fliC*Δ*siiD* compared to those infected with Δ*fliC* (Fig 6A). However, the enhancement of ASC oligomerization induced by Δ*fliC*Δ*siiD* was counteracted in *Nlrp3*^-/—^BMDMs. In addition, ASC speck formation was detected by indirect immunofluorescence to further confirm the inhibitory effect of SiiD on ASC oligomerization. As expected, a significantly higher quantity of ASC speck was observed in WT-, *Nlrc4*^-/—^, and *Casp1*^-/—^BMDMs infected with Δ*fliC*Δ*siiD* than with Δ*fliC*. No significant difference in the number of BMDMs with ASC speck between Δ*fliC* and Δ*fliC*Δ*siiD* group was observed in the absence of NLRP3 (Fig 6B and 6C). These results suggested that SiiD could prevent the formation of ASC pyroptosome via an NLRP3-dependent, NLRC4-, and Caspase-1-independent way.

### SiiD prevented mtROS generation to inhibit the formation of ASC pyroptosome

**Fig 7:**
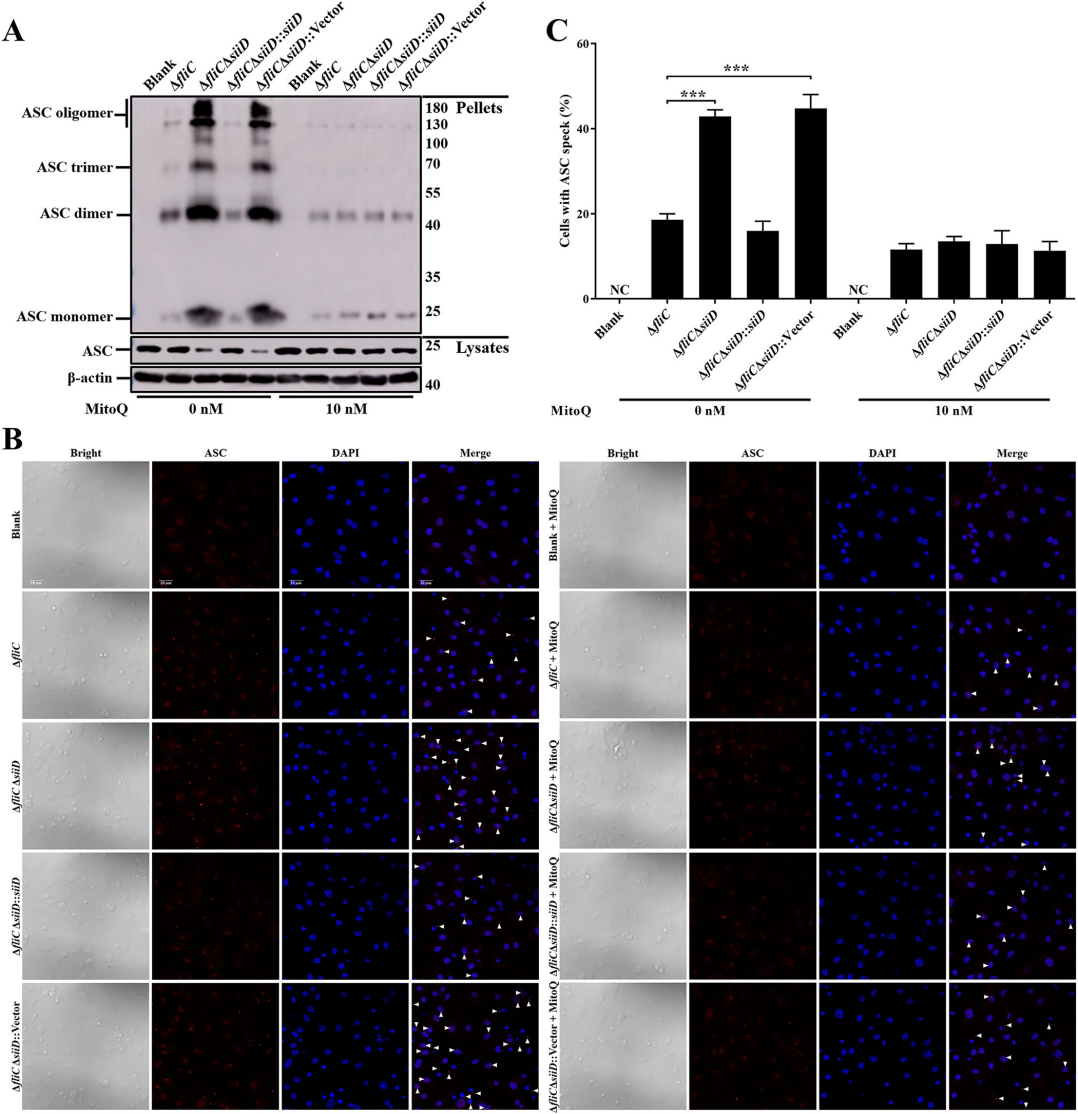
SiiD repressed the formation of mtROS dependent ASC pyroptosome. WT-BMDMs were primed with LPS (200 ng/mL) for 5 h, MitoQ (10 nM) or vehicle control (DMSO) was added to cells 1 h after LPS treatment. Then cells were infected with Δ*fliC*, Δ*fliC*Δ*siiD*, Δ*fliC*Δ*siiD*::*siiD*, or Δ*fliC*Δ*siiD*::Vector at an MOI of 50:1 for 4.5 h, uninfected cells were used as a negative control (Blank). (**A**) Cells were lysed and the pellets were subjected to cross-linking. The ASC oligomerization in the pellets and the total ASC in lysates as the input were examined by western blotting. β-actin was blotted as a loading control. Molecular mass markers in kDa are indicated on the right. (**B**) The formation of ASC specks (arrowheads) was detected by indirect immunofluorescence assay. ASC, red; DAPI, blue. Scale bar, 20 μm. (**C**) The percentages of cells with ASC speck. Approximately 200 cells were counted in each sample. Data are presented as mean ± SEM of triplicate samples per experimental condition from three independent experiments. ***p < 0.001, as measured by one-way ANOVA followed by Bonferroni’s multiple comparison test.

To further confirm whether the inhibitory effect of SiiD on ASC pyroptosome formation was associated with inhibiting mtROS generation, macrophages were pretreated with MitoQ before SE infection. In both MitoQ-untreated BMDMs and J774A.1 cells, ASC oligomerization was significantly induced by Δ*fliC*Δ*siiD* or Δ*fliC*Δ*siiD*::Vector compared to Δ*fliC* or Δ*fliC*Δ*siiD*::*siiD* (Figs 7A and S4), along with ASC speck formation (Figs 7B–7C and S4B–S4C). Whereas, the pretreatment with MitoQ markedly inhibited ASC oligomerization and ASC speck formation induced by Δ*fliC*Δ*siiD* or Δ*fliC*Δ*siiD*::Vector in both BMDMs and J774A.1 cells. There was no significant difference in the ability of inducing ASC pyroptosome formation between these indicated SE strains. Taken together, these results indicated that SiiD inhibited the activation of NLRP3 inflammasome via preventing mtROS generation-dependent ASC pyroptosome formation during SE infection.

### SiiD independently inhibited NLRP3 inflammasome activation by preventing mtROS-ASC Axis

**Fig 8:**
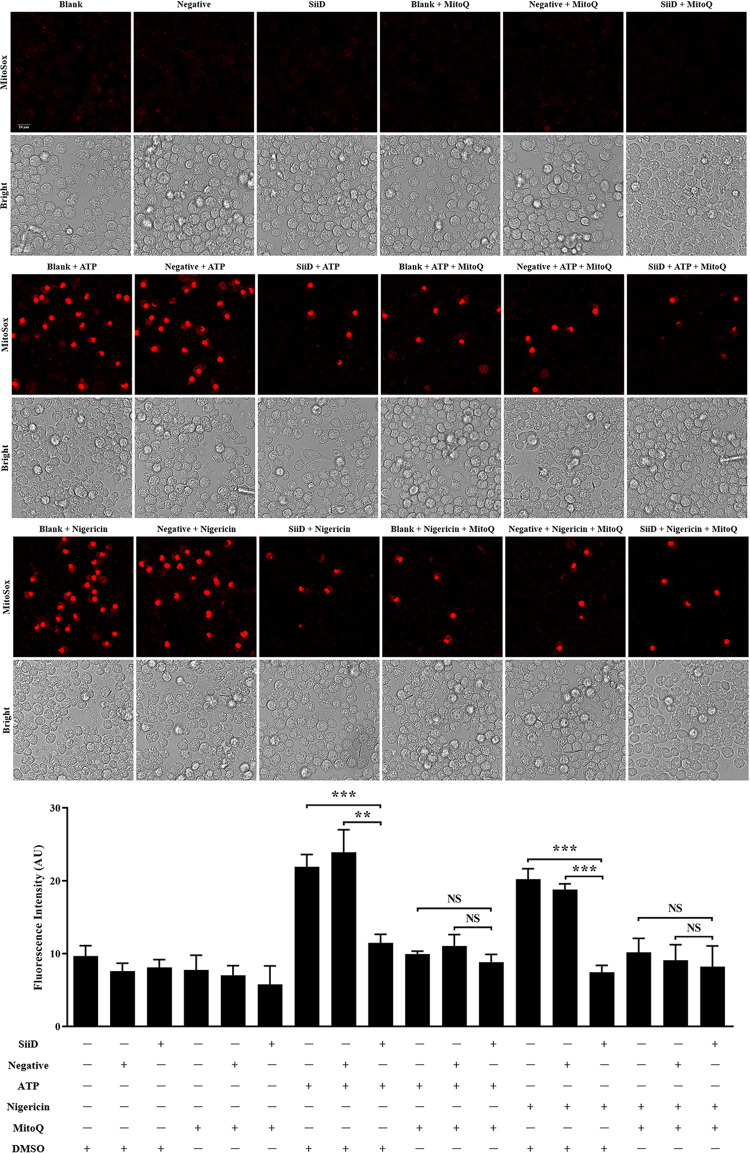
SiiD independently inhibited mtROS generation in macrophages. J774A.1 cells were transduced with Lv-SiiD or Lv-NC lentivirus, untreated cells were used as a negative control (Blank). Cells were primed with LPS (1 μg/mL) for 5 h, MitoQ (10 nM) or vehicle control (DMSO) was added to cells 1 h after LPS treatment. Then cells were stimulated with or without ATP (1.25 mM) or nigericin (10 μM) for 1 h. (**A**) Cells were loaded with MitoSOX Red (5 μM) for 30 min after stimulation. Production of mitochondrial superoxide in infected cells were assayed. Scale bar, 20 μm. (**B**) The mean MitoSOX red fluorescence was quantified using Application Suite software. Data are presented as mean ± SEM of triplicate samples per experimental condition from three independent experiments. **p < 0.01, ***p < 0.001; NS, not significant, as measured by one-way ANOVA followed by Bonferroni’s multiple comparison test.

SiiD has been shown to inhibit NLRP3 inflammasome activation independently. We expressed SiiD protein in J774A.1 cells to further confirm whether SiiD could independently prevent mtROS generation and ASC pyroptosome formation. After LPS priming, J774A.1 cells were stimulated with ATP or nigericin, and then transfected with lentivirus. Generation of mtROS was significantly decreased in SiiD-expressing J774A.1 cells, compared to the control cells and negative lentivirus transfected cells (Fig 8). Furthermore, we also found that the pretreatment of MitoQ significantly counteracted the enhancement of mtROS generation induced by ATP or nigericin. There was no significant difference in the level of mtROS generation among control cells, SiiD lentivirus, and negative lentivirus transfected cells. These results suggested that the SE T1SS protein SiiD could independently inhibit the generation of mtROS in macrophages.

**Fig 9:**
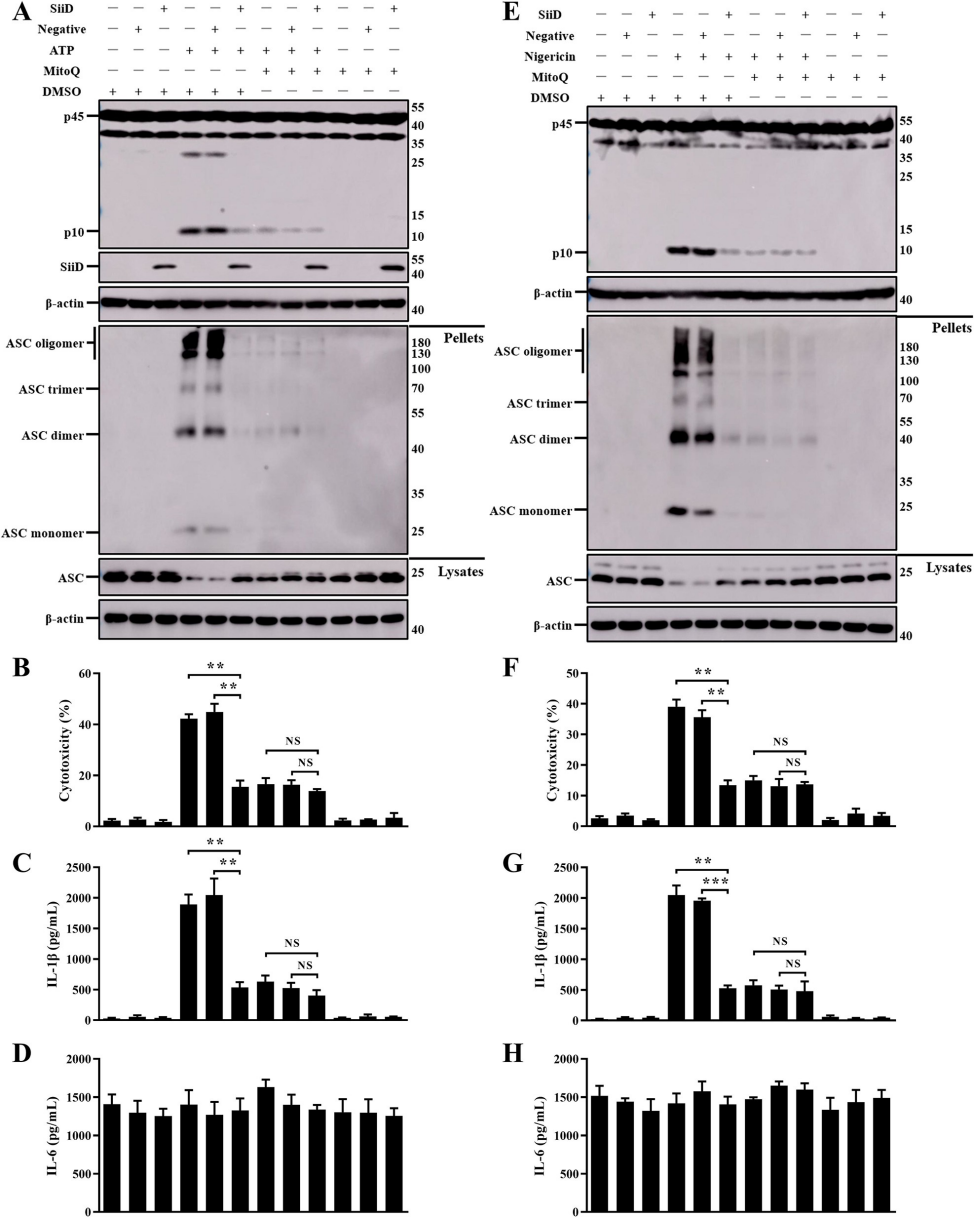
SiiD independently suppressed NLRP3 inflammasome activation by preventing mtROS-ASC signaling. J774A.1 cells were transduced with Lv-SiiD or Lv-NC lentivirus, untreated cells were used as a negative control (Blank). Cells were primed with LPS (1 μg/mL) for 5 h, MitoQ (10 nM) or vehicle control (DMSO) was added to cells 1 h after LPS treatment. The cells were then stimulated with or without ATP (1.25 mM) or nigericin (10 μM) for 1 h. (**A**, **E**) Cells were lysed and the pellets were subjected to cross-linking. The activation of Caspase-1 (p10), the expression of SiiD, the ASC oligomerization in the pellets, and the total ASC in lysates as the input were examined by western blotting. β-actin was blotted as a loading control. Molecular mass markers in kDa are indicated on the right. (**B**, **F**) Supernatants were analysed for cytotoxicity evaluated by LDH release. (**C**, **G**) IL-1β and (**D**, **H**) IL-6 secretion in supernatants were examined via ELISA. Data are presented as mean ± SEM of triplicate samples per experimental condition from three independent experiments. **p < 0.01, ***p < 0.001; NS, not significant, as measured by one-way ANOVA followed by Bonferroni’s multiple comparison test.

Similarly, our results also indicated that ASC speck formation was significantly lower in SiiD lentivirus transfected J774A.1 cells than in control cells and negative lentivirus transfected cells after stimulation with ATP or nigericin (Fig 9). Consistently, the addition of MitoQ largely decreased the formation of ASC pyroptosome in J774A.1 cells stimulated with ATP or nigericin. These results indicated that SiiD independently suppressed the formation of ASC pyroptosome via inhibiting mtROS generation.

**Fig 10:**
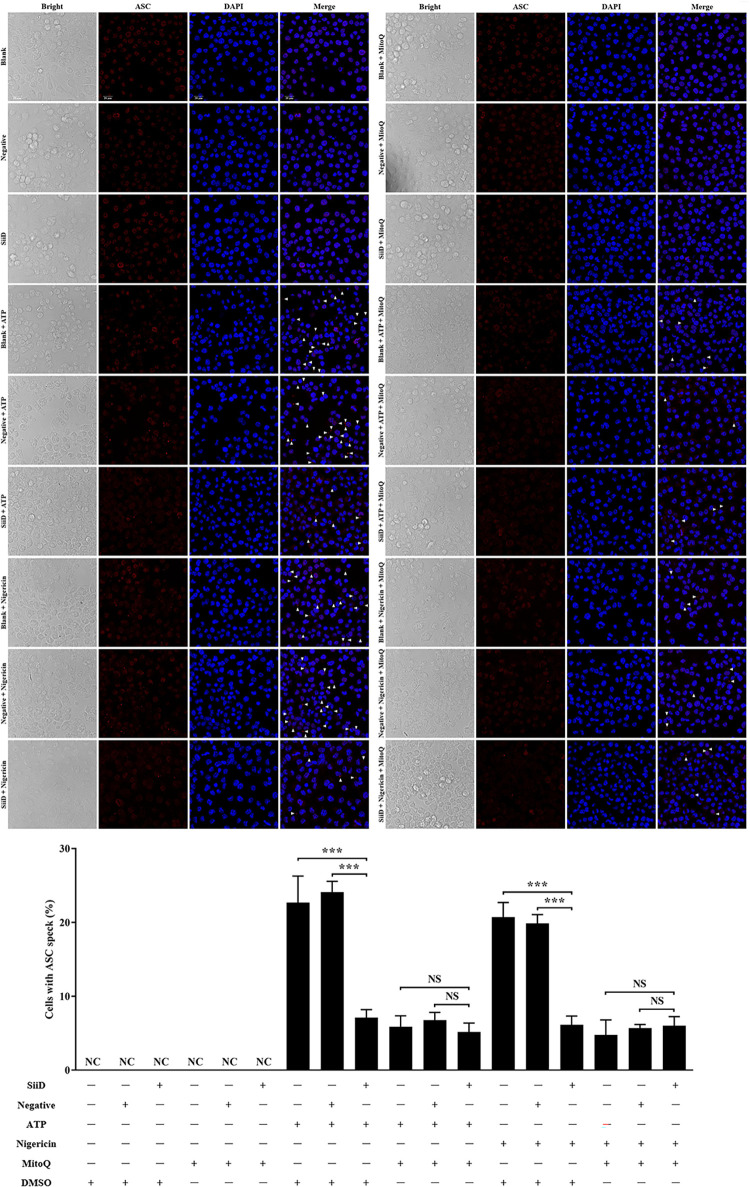
SiiD independently inhibited the formation of mtROS dependent ASC pyroptosome. J774A.1 cells were transduced with Lv-SiiD or Lv-NC lentivirus, untreated cells were used as a negative control (Blank). Cells were primed with LPS (1 μg/mL) for 5 h, MitoQ (10 nM) or vehicle control (DMSO) was added to cells 1 h after LPS treatment. Then cells were stimulated with or without ATP (1.25 mM) or nigericin (10 μM) for 1 h. (**A**) The formation of ASC specks (arrowheads) in infected BMDMs were detected by indirect immunofluorescence assay. ASC, red; DAPI, blue. Scale bar, 20 μm. (**B**) The percentages of cells with ASC speck. Approximately 200 cells were counted in each sample. Data are presented as mean ± SEM of triplicate samples per experimental condition from three independent experiments. ***p < 0.001; NS, not significant, as measured by unpaired *t* test.

Furthermore, it was demonstrated that the stimulation with ATP or nigericin strongly induced ASC oligomerization and NLRP3 inflammasome activation in J774A.1 cells [20]. Heterologous expression of SiiD significantly inhibited the increase in Caspase-1 activation, ASC oligomerization, macrophage death, and IL-1β secretion induced by ATP (Fig 10A–10C) or nigericin (Fig 10E–10G). Similarly, the inhibition of mtROS generation by MitoQ also prevented ASC oligomerization and NLRP3 inflammasome activation induced by ATP or nigericin in J774A.1 cells. However, the release of non-inflammasome cytokine IL-6 was not affected by SiiD or MitoQ (Fig 10D and 10H). Taken together, these results demonstrated that SiiD could independently inhibit the activation of NLRP3 inflammasome by negatively regulating the mtROS-ASC axis.

### SiiD repressed NLRP3 inflammasome-mediated clearance of SE *in vivo*

**Fig 11:**
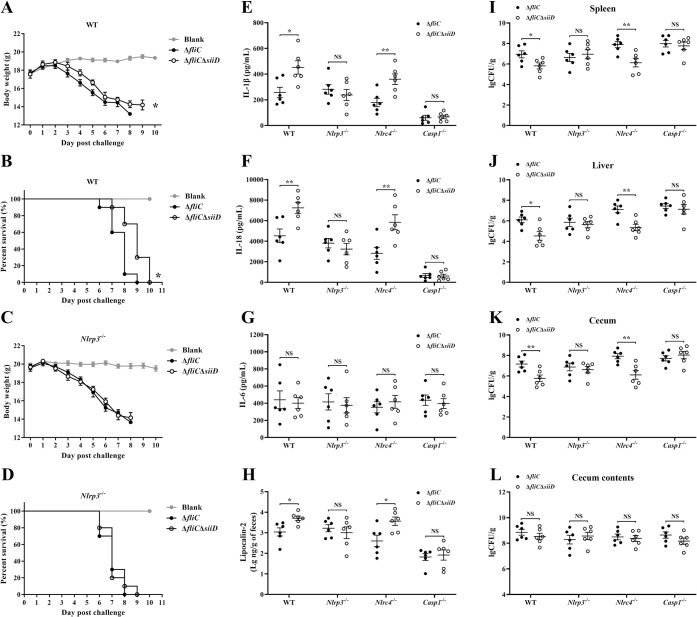
SiiD inhibited NLRP3 inflammasome mediated SE clearance *in vivo*. The WT- and *Nlrp3*^-/—^C57BL/6 mice were orally infected with Δ*fliC* or Δ*fliC*Δ*siiD* at a dose of 5 × 10^6^ CFU per mouse (n = 10). Uninfected mice were used as the negative control (Blank). The (**A**, **C**) body weight changes and (**B**, **D**) deaths of mice within 10 dpi were recorded by daily observation. *p < 0.05, as measured by two-way ANOVA for the group differences in weight of mice over time. *p < 0.05, as measured by log-rank (Mantel-Cox) test for the survival curve. The WT-, *Nlrp3*^-/—^, *Nlrc4*^-/—^, and *Casp1*^-/—^C57BL/6 mice were orally infected with Δ*fliC* or Δ*fliC*Δ*siiD* at a dose of 5 × 10^6^ CFU per mouse (n = 6). Serum (**E**) IL-1β, (**F**) IL-18, and (**G**) IL-6 levels in infected mice at 5 dpi were determined by ELISA. (**H**) Gut inflammation as measured in feces using lipocalin-2 ELISA. Data are presented as the mean ± SEM of log_10_ ng/g feces. *p < 0.05; NS, not significant, as measured by unpaired *t* test. The (**I**) spleen, (**J**) liver, (**K**) cecum, and (**L**) cecum contents homogenates were plated to determine the bacterial CFU per gram of organs at 5 dpi. Data are presented as mean ± SEM of log_10_ CFU/g. **p < 0.01, ***p < 0.001; NS, not significant, as measured by unpaired *t* test.

Inflammasome activation is an essential mechanism of the innate immune response in host defense against *Salmonella* infection. To further clarify the biological significance of SiiD inhibition of NLRP3 inflammasome activation during SE infection, WT- or *Nlrp3*^-/—^C57BL/6 mice were pretreated with streptomycin before orally infected with Δ*fliC* or Δ*fliC*Δ*siiD*. Weight changes and deaths of mice were recorded within 10 days post-infection (dpi). WT- or *Nlrp3*^-/—^mice infected with Δ*fliC* or Δ*fliC*Δ*siiD* suffered a significant loss of body weight compared to uninfected mice (Fig 11A and 11C). The body weight loss of Δ*fliC*Δ*siiD* infected WT-mice was significantly attenuated compared to the WT-mice infected with Δ*fliC* (Fig 11A). The death peak of WT-mice with 80% mortality appeared in 7–8 days after being infected with Δ*fliC*, while the death peak of WT-mice infected with Δ*fliC*Δ*siiD* was between 9–10 dpi (Fig 11B). In contrast, no significant differences were found in body weight loss and survival between *Nlrp3*^-/—^mice infected with Δ*fliC* and Δ*fliC*Δ*siiD*. These results suggested that SiiD promoted the virulence of SE in mice by interfering with NLRP3 signaling.

To determine the expression of the SE *siiABCDEF* operon *in vivo*, WT- and *Nlrp3*^-/—^C57BL/6 mice were orally infected with Δ*fliC* or Δ*fliC*Δ*siiD* at a dose of 5 × 10^6^ CFU per mouse. Bacteria were collected from the spleen, liver, cecum, and cecum contents at 5 dpi. The transcript levels of *siiABCDEF* were determined by qRT-PCR. The parental Δ*fliC* strain in the logarithmic phase *in vitro* was chosen as the calibrator. The expression level of *siiABCEF* in Δ*fliC*Δ*siiD* collected from the indicated organs was similar to that in Δ*fliC* (S5 Fig), indicating that the deletion of SiiD did not affect the expression of SE *siiABCDEF* operon *in vivo*. Furthermore, we found that the expression pattern of *siiABCDEF* was diverse in different organs, and the *siiABCDEF* operon was highly expressed in gut lumen. *siiCDF* were highly expressed in bacteria residing within the spleen, liver, cecum, and gut lumen compared to the SE strain *in vitro* (S5C, S5D, and S5F Fig). Markedly increased expression level of *siiA* (S5A Fig) and *siiE* (S5E Fig) was only found in SE strains colonized in the cecum and gut lumen, respectively.

WT-, *Nlrp3*^-/—^, *Nlrc4*^-/—^, and *Casp1*^-/—^C57BL/6 mice was used to further confirm the function of stronger NLRP3 inflammasome induced by Δ*fliC*Δ*siiD* in antibacterial defense *in vivo*. Mice serum, spleen, liver, cecum, cecum contents, and fresh fecal pellets were collected at 5 dpi. Bacterial colonization in mice tissues and the expression levels of inflammasome-dependent cytokines IL-1β and IL-18 in the serum were tested. Furthermore, detection of lipocalin-2 expression level in fecal was performed to evaluate the induction of gut inflammation, Compared to WT-mice, a significantly decreased levels of IL-1β, IL-18, and fecal lipocalin-2 were detected in infected *Casp1*^-/—^mice (Fig 11E, 11F, and 11H), suggesting that Caspase-1-dependent inflammasome was markedly activated in WT-mice after SE infection, accompanied by stronger gut inflammation. Consistently, the bacterial burdens were significantly decreased in the WT-mice spleen, liver and cecum after infection compared to *Casp1*^-/—^mice (Fig 11I–11K), indicating that the activation of Caspase-1-dependent inflammasome was essential for the clearance of SE in mice. Similar levels of bacterial colonization were detected in the cecum contents of each group of mice (Fig 11L).

Notably, the fecal lipocalin-2, serum IL-1β and IL-18 levels induced by Δ*fliC*Δ*siiD* in WT- and *Nlrc4*^-/—^mice were markedly higher than those induced by Δ*fliC* (Fig 11E, 11F, and 11H), coinciding with the significantly reduced bacterial colonization levels in the spleen, liver, and cecum of WT- and *Nlrc4*^-/—^mice infected with Δ*fliC*Δ*siiD* (Fig 11I–11K). However, no significant differences in inflammasome-dependent cytokines secretion, gut inflammation, or bacterial burdens were detected between *Nlrp3*^-/—^and *Casp1*^-/—^mice infected with Δ*fliC* or Δ*fliC*Δ*siiD*. In addition, *Nlrp3*^-/—^mice infected with Δ*fliC*Δ*siiD* exhibited increased bacterial burden in tissues and decreased fecal lipocalin-2, serum IL-1β and IL-18, in comparison with WT- or *Nlrc4*^-/—^mice infected with Δ*fliC*Δ*siiD* (Fig 11E–11K), indicating that NLRP3 plays a functionally essential role in response to Δ*fliC*Δ*siiD* infection. Of note, similar secretion level of IL-6 was found in mice sera between each group (Fig 11G). These results demonstrated that the inhibition of NLRP3 inflammasome activation mediated by SiiD contributed to bacterial evasion of the host immune clearance *in vivo*, which is essential for the pathogenicity of SE.

## Discussion

*Salmonella* infection can trigger the activation of NLRP3 and NLRC4 inflammasomes which are subsequently involved in pyroptosis and the clearance of bacteria *in vivo* [14]. Mice deficient in Caspase-1, IL-1β, or IL-18 reportedly succumbed to *Salmonella* infection earlier than WT-mice, with significantly higher bacterial burdens in the spleens, Peyer’s patches, and mesenteric lymph nodes [15], indicating that inflammasome activation is a key target for *Salmonella* immune evasion strategies. However, the mechanisms by which SE evades inflammasome activation have not been well elucidated. In the present study, a transposon insertion mutant library based on the SE strain Δ*fliC* was generated to identify potential factors essential for SE to evade inflammasome detection. Three genes (*siiD*, *sifA*, and *rcsD*), which were also required for long term persistence infection of *Salmonella*, were identified essential for inflammasome activation after two rounds of screening [21]. More importantly, we identified a novel factor, SiiD, which is a T1SS protein of SE that inhibits NLRP3 inflammasome activation. Our findings further confirmed that NLRP3 inflammasome could be a target for *Salmonella* to evade innate immune response.

*Salmonella* SPI-4 is a 27-kb region that carries six genes designated *siiABCDEF*. The periplasmic adaptor SiiD polymerizes to form a channel, bridging the outer membrane secretin SiiC and inner membrane transport ATPase SiiF to assemble type I secretion apparatus [22], which was similar to the T3SS-1 apparatus. The T3SS-1 rod protein PrgJ and needle protein PrgI polymerize to form a hollow tube that allows for the passage of secretory components [23]. However, the NLRC4 inflammasome could not detect PrgJ and PrgI proteins at the correct location within the T3SS apparatus. Unless T3SS exports excess rod monomers from the bacterial cytosol or rod monomers sloughed into the secretion channel, resulting in inadvertent translocation [24,25]. Here, we found that the transportation of SiiD into host cells may also be mediated by inadvertent translocation. While the transport of SiiD proteins was obviously beneficial to the bacteria, as SiiD promoted SE to evade recognition by the NLRP3 inflammasome, and facilitated SE replication and survival within the infected host.

A previous study showed that loss of SPI-4 attenuated the oral virulence of ST and SE in mice [26], which was consistent with our findings that SiiD contributed to the oral virulence of SE. Our results showed that the deficiency of SiiD did not affect the normal expression of T1SS and T3SS-1 in SE strains collected from infected macrophages, which was consistent with a previous study that mutations in SPI-4 did not affect the expression or secretion of T3SS-1-dependent *Salmonella* proteins [27]. These results also suggested that SiiD specifically repressed the activation of inflammasome. Previous studies have shown that both T3SS-1 and T1SS were crucial factors for *Salmonella* pathogenesis, which were tightly regulated by the global regulators SirA and HilA in the late logarithmic phase [28]. The cooperative activity of SPI-1 and SPI-4 was required for *Salmonella* to destroy the epithelial barrier and invade polarized epithelial cells from the apical side [18]. Furthermore, T3SS-1 has also been reported to be essential for *Salmonella* to invade intestinal epithelial cells [29]. These results may explain why the intracellular translocation of SiiD mediated by T1SS was markedly defective in the absence of T3SS-1.

A recent study reported that the *siiABCDEF* promoter was more highly expressed in ST residing within the cytosol as compared to the bacteria hiding in SCV [30]. This may be related to the presence of inflammasomes in the cytosol, which could recognize *Salmonella* and trigger innate immune responses. In this study, we found that the mRNA expression of T1SS apparatus (*siiC*, *siiD*, and *siiF*) were highly induced in SE strains residing within spleen, liver, gut lumen, and cecum. Similarly, SiiC, SiiD, and SiiF of ST were also found to be highly expressed in cytosol of infected host cells as compared to SCV [30]. The high expression of T1SS may be essential for protecting *Salmonella* against the harmful environmental factors in the cytosol. Previous studies have reported that *Salmonella* T1SS was required for the invasion of polarized epithelial cells, and entirely redundant for the invasion of non-polarized epithelial cells [18], whereas the T1SS was not required for invasion or persistence infection in macrophages [27]. In contrast, a ST mutant strain with a transposon insertion in SPI-4 was reported to be deficient in its ability to survive in murine peritoneal macrophages [31,32]. Subsequent studies have revealed that SPI-4 plays an important role in the survival of SE in chicken macrophages [33]. Notably, we identified a novel function of T1SS that could suppress NLRP3 inflammasome activation in immune cells. Our results indicated that the function of T1SS was not limited to the gut, and that T1SS still played an important role in promoting bacterial evasion of immune clearance at systemic sites.

Unlike the NLRC4 inflammasome, the NLRP3 inflammasome responds to a variety of chemically and physically distinct active signals, including whole pathogens, diverse PAMPs, DAMPs, and environmental irritants [19]. Multiple upstream signals, including K^+^ or Cl^−^ efflux, Ca^2+^ flux, lysosomal disruption, metabolic changes, trans-Golgi disassembly, and mitochondrial dysfunction [2,34–37], implicated in triggering NLRP3 inflammasome activation, were always accompanied by the generation of intracellular ROS. Previous studies have demonstrated that the generation of mtROS was required for the priming of NLRP3, whereas the deubiquitination of NLRP3 mediated by mtROS was essential for NLRP3 in response to LPS stimulation [38]. A recent study revealed that phosphoinositide 3-kinase (PI3K) activated by mtROS allowed mitochondria to transfer from bone marrow stromal cells into hematopoietic stem cells, which was critical for the clearance of *Salmonella* infection [39]. MCLK1 (clock abnormality-1) was a mitochondrial hydroxylase required for ubiquinone biosynthesis, and loss of MCLK1 promoted the generation of mtROS, resulting in increased expression of HIF-1α and TNF-α in macrophages, which was beneficial for the clearance of SE [40]. Here, we report that SE utilized the T1SS protein, SiiD, to prevent host NLRP3 inflammasome signaling and evade host immune clearance by repressing the generation of mtROS. Consistently, TCA cycle enzymes of ST involved in the suppression of mtROS-dependent NLRP3 inflammasome activation, which was essential for ST oral virulence in mice [41]. MitoQ pretreatment counteracted the enhancement of NLRP3 inflammasome activation mediated by mtROS in macrophages infected with both SiiD and TCA enzyme-deficient *Salmonella* strains. The assembly of ASC pyroptosomes by oligomerized ASC dimers has been previously reported to be a key event in NLRP3 inflammasome activation [42]. Recently, mtROS was found to be involved in omega-class glutathione transferase 1-mediated ASC deglutathionylation, which was required for ASC pyroptosome formation and NLRP3 inflammasome activation [43], indicating that the mtROS-ASC pathway played a critical role in NLRP3 inflammasome activation. In this study, we confirmed the involvement of intracellular mtROS in modulating NLRP3 inflammasome activation, and further revealed that ASC oligomerization was the downstream target of mtROS signaling. More importantly, we found that the SE T1SS protein SiiD targeted intracellular mtROS to depress ASC pyroptosome formation and subsequently NLRP3 inflammasome activation. Similarly, the bacterial catalytic subunit α of the enzyme nitrate reductase promoted the production of NO during *Brucella abortus* infection, which was required to prevent the accumulation of mtROS and subsequent ASC pyroptosome-mediated NLRP3 inflammasome activation [44,45]. These studies suggest that the mtROS-ASC axis, as an essential signal for NLRP3 inflammasome activation, might become a non-negligible target for bacteria to repress inflammasome detection and evade host immune clearance.

Neither ASC nor NLRP3 played a significant role in host defense against ST infection, and IL-18 release was unaffected in infected *Nlrp3*^-/—^mice [46]. NLRP3 was dispensable for ST clearance in the cecum and mesenteric lymph nodes, even in the absence of NLRC4 [47]. Similar results were obtained in this study, and no significant difference in bacterial colonization was found between WT- and *Nlrp3*^-/—^mice after infection, as well as the serum IL-1β and IL-18 expression levels. Based on in-depth studies, the inability of NLRP3 to mediate *Salmonella* clearance could be attributed to the multiple strategies implemented by *Salmonella* to inhibit NLRP3 inflammasome activation during infection [41,48]. Here, we identified a new factor, SiiD, that promoted dent NLRP3 inflammasome activation and in turn promoted immune evasion. SPI-4 was required for ST to colonize calf intestines [49], but mutants with transposon insertions in SPI-4 genes were not attenuated in intestinal colonization in chickens [27] or pigs [50]. Here, we found that the deletion of *siiD* did not affect SE colonization in the mice gut lumen. These data indicated that *Salmonella* may have different intestinal colonization strategies in different hosts.

Notably, despite significantly reduced bacterial colonization in the organs, SiiD-deficient SE strains still induced remarkably higher levels of serum IL-1β and IL-18 compared to the parental strain, indicating that SE lacking SiiD induced intense inflammasome activation *in vivo*. Our findings might explain why *Salmonella* was preferentially associated with anti-inflammatory/M2 macrophages at later stages of long-term infection [51]. It was obvious that the bacteria choose M2 macrophages, which were fueled with oxidative phosphorylation, rather than M1 macrophages, which were fueled with glycolysis, as this effectively avoided the activation of NLRP3 inflammasome. Thus, it is likely that the NLRP3 inflammasome detected cell stress associated with bacterial intracellular oxidative metabolism and therefore presents a logical target for immune evasion and suppression by SE. Notably, the NLRP3 inflammasome was identified as a crucial element to trigger T cell responses in the adjuvant effect of aluminum adjuvants for human vaccines, whereas innate inflammasome signals could direct a humoral adaptive immune response [52]. Therefore, in addition to revealing a novel aspect of interactions between *Salmonella* and inflammasomes, this study might also provide a potential strategy against salmonellosis, by triggering robust NLRP3 inflammasome activation to induce adaptive cellular immune responses.

Fecal lipocalin-2 levels were shown to correlate with varying degrees of inflammation restricted to the intestine [53]. Lipocalin-2 was found to exert anti-bacterial activity upon binding of microbial siderophore enterobactin [54]. Divalent metal transporter 1 (DMT1) in mice macrophages was essential for the expression of lipocalin-2, and DMT1-deficient mice exhibited increased vulnerability to *Salmonella*, with reduced lipocalin-2 expression and a higher bacterial burden in tissues [55]. Consistently, we found that the SE Δ*fliC* strain utilized SiiD to repress NLRP3 inflammasome activation *in vivo*, resulting in the increased bacterial colonization in mice tissues and significantly decreased fecal lipocalin-2 level as compared to SiiD-deficient SE strain. Our results further confirmed that the lipocalin-2 related to the colonization of SE in mice tissues. *Salmonella* was found to secrete another modified form of enterobactin, termed salmochelins. The production of salmochelins was associated with the resistance to lipocalin-2 and allowed bacterial growth in the inflamed intestine [56]. This may explain why the higher lipocalin-2 level in the lumen of the inflamed intestine did not affect colonization of the SiiD deletion mutant strain. A recent study showed that lipocalin-2 could directly up-regulate the assembly of the NLRP3 inflammasome and the expression of IL-1β via NF-κB activation in LPS-stimulated macrophages [57]. The increased circulatory lipocalin-2 promoted the secretion of a high mobility group box 1 (HMGB1) that subsequently induces oxidative stress and NLRP3 inflammasome activation on brain cells [58]. Lipocalin-2 knockout mice exhibited less pressure overload-induced NLRP3 inflammasome activation than WT mice [59]. These results suggested that although *Salmonella* was conferred lipocalin-2 resistance, but lipocalin-2 may still contribute to the clearance of bacteria from organs by activating the NLRP3 inflammasome.

**Fig 12:**
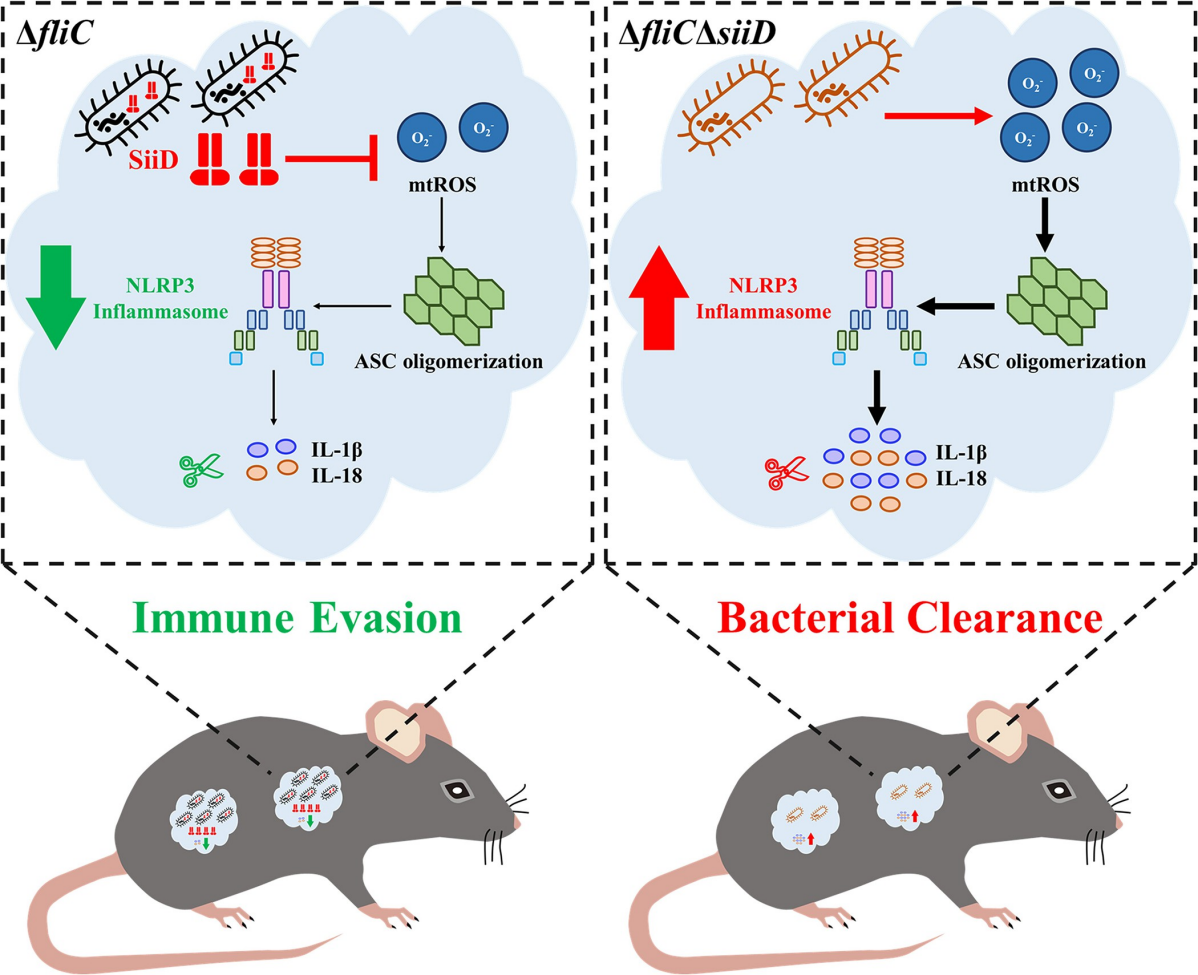
Inhibition of mtROS-ASC-dependent NLRP3 inflammasome activation mediated by SE T1SS protein SiiD contributed to bacterial immune evasion. *Salmonella* Enteritidis invading into host cells released SiiD via T1SS to inhibit intracellular mtROS production, and subsequently inhibited ASC pyroptosome formation, and finally repressed NLRP3 inflammasome activation and expression of IL-1β and IL-18, which contributed to bacterial immune evasion.

In summary, various bacterial T3SS and T4SS effectors have been identified to regulate inflammasome activation [60–62] but the contribution of bacterial T1SS to pathogenicity is not well understood. To our knowledge, this study is the first to identify a bacterial T1SS protein that affects the activation of the NLRP3 inflammasome. SiiD was localized in the membrane fraction after being translocated into host cells by SE in a T1SS-dependent and partially T3SS-1-dependent way. Our data further revealed that SE utilized the T1SS protein SiiD to inhibit mtROS generation-dependent ASC oligomerization and in turn repressed NLRP3 inflammasome activation (Fig 12). More importantly, SiiD was required for SE to reduce the gut inflammation and evade host immune clearance mediated by NLRP3 inflammasome *in vivo*, which might be essential for bacterial colonization during long-term infection. Our study provided a link between the inhibition of NLRP3 inflammasome activation and immune escape of bacteria, supported the contributions of inflammasome activation modulation to long-term SE persistence, and increased our knowledge of the complex host-pathogen interactions.

## Material and methods

### Ethics statement

All animal experiments were approved by the Animal Welfare and Ethics Committees of Yangzhou University and complied with the guidelines of the Institutional Administrative Committee and Ethics Committee of Laboratory Animals (IACUC license number: YZUDWLL-201811-001). All the animals were humanely handled.

### Bacterial strains and transposon mutant library

The WT SE strain C50336 was obtained from the National Institute for the Control of Pharmaceutical and Biological Products (Beijing, China). All bacterial strains and plasmids used in this study are listed in S1 Table. To exclude possible interference by flagellin, a strong activator of NLRC4 inflammasome, all targeted deletion strains and transposon mutants used in this study were constructed based on the SE flagellin deletion strain Δ*fliC*. The gene deletion mutant strains were constructed by double exchange of homologous recombination using pDM4 plasmid as described previously [63]. Plasmid pBAD33 was employed for complementation of target genes. All the primers used for the construction of recombinant bacteria are listed in S2 Table.

The generation of *mariner*-based transposon mutant library was achieved by using the delivery plasmid pSC189 to randomly insert the TnpSC189 transposon into Δ*fliC* chromosome as described previously [63]. Two-round semi-arbitrary PCR was used to determine the location of the transposon insertion as described previously [63]. The PCR primers and sequencing primer are listed in S2 Table. The sequences were compared with the GenBank DNA sequence database using the BLASTX program.

### Mice and cell culture

Wild type (WT) specific pathogen-free (SPF) female C57BL/6 mice (age, 6–8 weeks; body weight, 20 ± 2 g) were obtained from the Comparative Medical Center of Yangzhou University (Yangzhou, China). The following knock-out mice used in this study were generated based on the C57BL/6 mice. SPF NLRP3-deficient (*Nlrp3*^-/-^) mice, NLRC4-deficient (*Nlrc4*^-/-^) mice, and Caspase-1-deficient (*Casp1*^-/-^) mice were purchased from Shanghai Model Organisms Center, Inc. (Shanghai, China) and bred under SPF conditions, in the mouse isolators (Suzhou monkey animal experiment equipment Technology Co. Ltd., Suzhou, China).

Mice bone marrow-derived macrophages (BMDMs) were obtained from mice bone marrow of the tibia and femur as described previously [63]. Bone marrow cells were grown at 37°C with 5% CO_2_ in Dulbecco’s modified Eagle’s medium (DMEM, Gibco, Grand Island, NY, USA) containing 10% (v/v) fetal bovine serum (FBS, Gibco), 100 U/mL penicillin, 100 μg/mL streptomycin (Gibco), and 25 ng/mL macrophage colony-stimulating factor (M-CSF, PeproTech, Rocky Hill, NJ, USA) for 6–7 days to differentiate into mature BMDMs. J774A.1 and HeLa cells were purchased from American Tissue Culture Collection (ATCC, Manassas, VA, USA) and cultured in complete DMEM supplemented with 10% FBS, penicillin (100 U/mL), and streptomycin (100 μg/mL). Cell viability and number were determined by trypan blue exclusion assays.

### *Salmonella* infections

J774A.1 cells or mice BMDMs were seeded at a concentration of 1 × 10^5^, 2 × 10^5^, 5 × 10^5^, or 1 × 10^6^ cells per well into 48-, 24-, 12-, or 6-well plates and cultured overnight. Overnight SE cultures were diluted 1:100 and cultured for another 3 h to an OD_600_ of 0.7 to induce SPI-1 expression. Cells were washed with Dulbecco’s PBS (DPBS, Gibco); J774A.1 cells were then pre-treated with 1 μg/mL LPS (Sigma-Aldrich, St. Louis, MO, USA) diluted in Opti-MEM (Gibco) for 5 h and BMDMs were pre-treated with 200 ng/mL LPS. After washing twice with sterile PBS, bacteria strains were added to the *Nlrc4*^-/—^BMDMs at an MOI of 100:1, the WT-, *Nlrp3*^-/—^, and *Casp1*^-/—^BMDMs were infected with bacteria at an MOI of 50:1. Cells were incubated at 37°C for 1.5 h, gentamicin was added to the cells at a final concentration of 50 μg/mL, and the cells were incubated for an additional 3 h. For MitoQ treatment, 10 μM MitoQ (MedChem Express, Monmouth Junction, NJ, USA) or vehicle control (DMSO) was added to cells 1 h after LPS treatment and remained for the duration of the experiment.

SPF C57BL/6 mice (6–8 weeks old) were randomly assigned to each group. Mice were fasted and water-deprived for 4 h before being gavaged with 7.5 mg of streptomycin. Following that, the mice were fasted and water-deprived for another 4 h before resuming diet for 20 h. Each group was orally infected with 300 μL of Δ*fliC* or Δ*fliC*Δ*siiD* diluted in PBS at a dose of 5 × 10^6^ CFU per mouse. The control mice received 300 μL of PBS via the same route. The body weight changes and deaths of mice within 10 dpi were recorded by daily observation. For testing the activation of inflammasome *in vivo*, the mice were sacrificed at 5 dpi and blood, spleen, liver, cecum, cecum contents, and fresh fecal pellets were collected. The expression levels of IL-1β, IL-18, and IL-6 in mice sera were measured by ELISA. To verify gut inflammation, fecal lipocalin-2 was detected as follows: fecal pellets were weighed, homogenised, and diluted in sterile PBS. The dilutions were then analyzed by ELISA. Mice tissues and cecum contents were weighed and homogenised in 1 mL of PBS. Serial 10-fold dilutions of tissue homogenates (100 μL each) were plated on XLT4 agar and incubated at 37°C for 12 h. Bacteria were counted and their numbers were expressed as log_10_ CFU/g.

### Cytotoxicity and ELISA assays

J774A.1 cells or BMDMs cultured in 48-well plates were infected with SE strains as described above, the supernatants were harvested at 4.5 h after infection. Cytotoxicity was quantified using the LDH Cytotoxicity Assay Kit (Beyotime Biotechnology Co. Ltd., Haimen, China) according to the manufacturer’s instructions. Expression of the cytokines IL-1β, IL-18, and IL-6 in supernatants and mice sera was quantified by ELISA using Mouse IL-1 beta/IL-1F2 DuoSet ELISA, Mouse IL-18 DuoSet ELISA, and Mouse IL-6 DuoSet ELISA (R&D Systems, Minneapolis, MN, USA) according to the manufacturer’s manual. Fecal lipocalin-2 was quantified by ELISA using Mouse NGAL/Lipocalin-2 ELISA Kit (Beyotime) according to the manufacturer’s instructions.

### Western blotting assays

J774A.1 cells or BMDMs cultured in 12-well plates were infected with SE strains as described above. After harvesting the supernatants, the cells were lysed with Cell lysis buffer for Western and IP (Beyotime). The supernatant and lysate were centrifuged at 2000 rpm for 5 min and mixed. An equal volume of methanol and a 0.25 volume of chloroform were added. The mixture was centrifuged at 12000 rpm for 5 min after vortexing. An equal volume of methanol was added after removing the upper aqueous phase. The supernatant was removed after centrifugation. The protein pellets were dried at 55°C for 5 min, resuspended with 1 × SDS-PAGE Sample Loading Buffer (Beyotime), and boiled for 10 min at 95°C. The protein samples were loaded onto 15% Tris-glycine gels, then transferred to nitrocellulose membranes and blotted with anti-Caspase-1 p10 antibody, anti-NLRP3/NALP3 antibody, anti-ASC antibody (AdipoGen, San Diego, CA, USA), anti-NLRC4 antibody, anti-DnaK antibody (Abcam, Cambridge, UK), anti-IL-1β antibody (Cell Signaling Technology, Danvers, MA, USA), anti-β-actin antibody (Sigma-Aldrich), anti-HA antibody, anti β-tubulin antibody (HuaAn biotechnology Co. Ltd., Hangzhou, China), and anti-Calnexin antibody (Beyotime). Secondary antibody was goat anti-mouse IgG-HRP or goat anti-rabbit IgG-HRP (Cell Signaling Technology). The nitrocellulose membranes were exposed with Amersham Imager 600 Imaging System (GE Healthcare Life Sciences, Pittsburgh, PA, USA) by using ECL chemiluminescence substrate (Thermo Scientific, Waltham, MA, USA).

### Quantitative real-time PCR (qRT-PCR) assays

J774A.1 cells cultured in 6-well plates were infected with the SE strains as described above. The cells were then washed thrice with DPBS and lysed with precooled PBS containing 0.1% Triton X-100 for 5 min. The cell lysates were centrifuged at 3200 × g for 10 min, and the pellets were harvested for subsequent bacterial RNA isolation using a TRNzol Universal total RNA Extraction Kit (Tiangen Biotech Co. Ltd., Beijing, China) according to the manufacturer’s instructions. Mice organs were harvested and homogenized as described above. Larger cell debris were removed from the homogenized samples by centrifugation at 100 × g for 5 min. Bacteria were collected by centrifugation for 10 min at 3200 × g, and then total RNA was extracted from bacterial pellets. RNA of the indicated SE strains was quantified using a One drop spectrophotometer (Wins Technology Co. Ltd., Nanjing, China). HiScript III RT SuperMix for qPCR (Vazyme Biotechnology Co. Ltd., Nanjing, China) was used to remove genomic DNA (gDNA) and reverse transcribe the RNA into cDNA. The reaction was performed in a total volume of 20 μL, containing 1 μg of total RNA, 4 μL of 4 × gDNA wiper Mix, and reactions were brought up to 20 μL with DEPC-treated water. The mixture was then incubated at 42°C for 2 min. Next, 5 μL of 5 × HiScript III qRT SuperMix was added, the mixture was incubated at 37°C for 15 min, followed by incubation at 85°C for 5 s, subsequently stored at −20°C until further use.

The Applied Biosystems QuantStudio 6 Flex Real-Time PCR System (Applied Biosystems, Foster City, CA, USA) was used to measure the mRNA expression levels of *siiABCDEF* and T3SS-1 apparatus (*prgHIJK*, *spaPQRS*, *sipBCD*, and *invAG*) and the internal control gene, the SE gyrase B (*gyrB*) gene. Primers were designed using Primer Express software v3.0 (Applied Biosystems, Carlsbad, CA, USA) and were listed in S3 Table. The qRT-PCR reaction was performed in a total volume of 20 μL, containing 200 ng of cDNA, 10 μL of 2 × AceQ Universal SYBR qPCR Master Mix (Vazyme), 0.6 μL each of 10 μM forward and reverse primers, and 6.8 μL RNase-free water. The comparative threshold cycle (2^–ΔΔC(T)^ method) was used to calculate relative concentrations. All qRT-PCR reactions were performed in triplicate.

### Lentivirus infection assays

Lentiviral vectors pGLV5-*siiD* (EF-1aF/GFP&Puro) were designed, constructed, amplified, and purified by GenePharma (Shanghai, China). The SiiD lentivirus Lv-SiiD and negative control lentivirus Lv-NC at a titer of approximately 1 × 10^9^ infectious units/mL were provided by GenePharma. Lentivirus was diluted 10-fold with complete DMEM and used to infect J774A.1 cells cultured in plates. The cells were incubated at 37°C with polybrene (GenePharma, final concentration of 5 μg/mL) for 24 h. After culturing in fresh complete DMEM for another 48 h, the cells were pre-treated with LPS and stimulated with the indicated concentration of ATP (1.25 mM, Thermo Scientific), or nigericin (10 μM, AdipoGen) for 1 h, or MSU crystals (200 μg/mL, AdipoGen) for 6 h. The supernatants and cell lysates were collected for western blotting or ELISA as described above.

### SiiD translocation assays

For FRET assays, SiiD was fused with TEM-1 β-lactamase by employing the plasmid pCX340 as described previously [63]. HeLa cells were seeded at a concentration of 2 × 10^5^ cells per well into black, clear-bottomed 24-well plates (Cellvis, Mountain View, CA, USA) and cultured overnight. SE strains bearing plasmid pCX340 were added to the cells at an MOI of 100:1. Bacteria were removed after infection for 3 h, and the cells were incubated for an additional 5 h. Then cells were loaded with CCF2-AM (Invitrogen, Carlsbad, CA, USA) for 2 h, and images were acquired using a Leica confocal microscope (Leica Microsystems, Wetzlar, Germany).

For subcellular localization assays, plasmid pBAD33 was used for the expression of SiiD-HA fusion protein. HeLa cells were seeded at a concentration of 1 × 10^7^ cells per 10 cm dish and cultured overnight. Cells were infected with SE strains bearing pBAD33-*siiD*-HA for 8 h as described above. Then cells were resuspended and lysed with precooled cell lysis buffer (250 mM sucrose, 3 mM imidazole, 0.5 mM EDTA, and a protease inhibitor mixture [pH 7.6]) by using a 1 mL syringe. The lysate was centrifuged at 5000 rpm for 10 min at 4°C. The sediment was set as Pellets fraction, and the supernatant was ultracentrifuged at 40000 × g for 30 min at 4°C. The supernatant (Cytosol fraction), sediment (Membrane fraction), and Pellets fraction were collected and mixed with SDS-PAGE Sample Loading Buffer (Beyotime) for western blotting assays.

### Indirect immunofluorescence assays

HeLa cells, J774A.1 cells, or BMDMs were seeded into 24-well chamber slides and infected with SE strains or lentivirus as described above. Then cells were fixed with 4% paraformaldehyde for 15 min. After washing three times with TBSTx (150 mM NaCl, 20 mM Tris-base, adjusting pH to 7.6 with HCl, adding 1% Triton X-100), cells were blocked with TBSTx containing 5% BSA for 1 h. Cells were incubated with anti-ASC antibody or anti-HA antibody, followed by Alexa Fluor 488-Labeled Goat Anti-Rabbit IgG or Alexa Fluor 555-Labeled Donkey Anti-Mouse IgG (Beyotime) and DAPI (Thermo Scientific). Images were acquired via Leica confocal microscope.

### Measurement of lysosomal disruption, K^+^ and Ca^2+^ fluxes

J774A.1 cells cultured in black, clear-bottomed 24-well plates were infected with the SE strains as described above. Cells in positive control group were treated with the lysosomal rupture inducer LLOMe (1 mM, 2 h). The cells were treated with 1 mg/mL acridine orange (AO) (Sigma-Aldrich) for 15 min. The residual dye was removed by washing three times with DPBS, and then fresh Opti-MEM was added to the plates. AO fluoresces red in the lysosomes and green in the cytosol. Images were acquired using a Leica confocal microscope and the mean AO red fluorescence was quantified using the Application Suite software (Leica). J774A.1 cells cultured in 6-well plates were infected with SE strains as the described above. The supernatants were harvested at 4.5 h after infection. The cells were washed with DPBS and lysed with 10% (v/v) nitric acid solution for 15 min. Concentration of K^+^ and Ca^2+^ in supernatants and cell lysates were quantified by PinAAcle 900Z graphite furnace atomic absorption spectrometer (PerkinElmer, Waltham, MA, USA).

### Measurement of mtROS generation

J774A.1 cells or BMDMs cultured in black, clear-bottomed 24-well plates were infected with SE strains or lentivirus as described above. Cells were loaded with 5 μM MitoSOX Red (Invitrogen) for 30 min. Residual dye was removed by washing three times with DPBS and then fresh Opti-MEM was added into the plates. Images were acquired via Leica confocal microscope and the mean MitoSOX red fluorescence was quantified using Application Suite software (Leica).

### ASC pyrotosomes detection

J774A.1 cells or BMDMs cultured in 6-well plates were infected with SE strains or lentivirus as described above. Cells were then lysed with precooled PBS containing 0.5% Triton X-100 for 30 min at 4°C. Cell lysates were centrifuged at 900 × g for 5 min at 4°C to remove cell debris. The pellets were washed twice with PBS and resuspended in 200 μL PBS. The pellets were then cross-linked with fresh 4 mM DSS (Thermo Scientific) for 30 min at 37°C and pelleted by centrifugation at 6800 × g for 20 min at 4°C. The cells lysates and cross-linked pellets were collected for ASC oligomerization assay by western blotting.

### Statistical analysis

The Z score was calculated for each well in a 48-well plate as described previously [63]. Briefly, the Z score of each given well was calculated by subtracting the mean cytotoxicity value of the wells on that plate from the cytotoxicity value of the given well and dividing by the standard deviation value for all of the plate wells. A Z score ≤ -2 or ≥ 2 was considered significant. All data are presented as mean ± standard error (SEM) of triplicate samples per experimental condition from three independent experiments using GraphPad Prism 5 software (La Jolla, USA). To detect significant differences between the experimental groups, the unpaired *t* test was used for two groups, a one-way analysis of variance (ANOVA) followed by Bonferroni’s multiple comparison test were used for multiple groups. Group differences in the weights of infected mice over time were analyzed by a two-way ANOVA. Log-rank (Mantel-Cox) test (conservative) was used to detect significant differences between survival curves of mice groups. Statistical significance was determined at p values of <0.05 (*), <0.01(**), or <0.001 (***).

## Supporting information

S1 Table Bacterial strains and plasmids used in this study. (DOCX)

S2 Table Primers used in this study. (DOCX)

S3 Table Primers used for qRT-PCR in this study. (DOCX)

S1 Fig Deletion of SiiD did not affect the growth characteristics and invasiveness of SE. (**A**) Distribution of Z score data of cytotoxicity levels induced by 3409 Δ*fliC* transposon mutants. The Z score was calculated for each well in a 48-well cell plate, and a Z score ≤ -2 or ≥ 2 was considered significant. (**B**) The transposon insertion sites of each candidate transposon mutants. Horizontal arrows indicate the direction of gene expression. Candidate gene, green; Upstream gene, blue; Downstream gene, brown. The numbers represent the initial or terminal position in the SE genome of each gene. The red vertical arrows represent the transposon insertion site of each candidate transposon mutants. (**C**) Growth curves of Δ*fliC*, Δ*fliC*Δ*siiD*, Δ*fliC*Δ*siiD*::*siiD*, and Δ*fliC*Δ*siiD*::Vector. Bacteria were grown in liquid LB medium at 37°C for 12 h with agitation, and the OD_600_ values of triplicate cultures in LB medium were determined in 1-h intervals. (**D**) J774A.1 cells were infected with Δ*fliC*, Δ*fliC*Δ*siiD*, Δ*fliC*Δ*siiD*::*siiD*, or Δ*fliC*Δ*siiD*::Vector at an MOI of 100:1 for 1.5 h. Bacterial lysates were plated to determine the invasiveness of SE strains. (**E, F**) J774A.1 cells were infected with the indicated SE strains at an MOI of 100:1 for 4.5 h. Bacterial lysates were plated to determine the invasiveness of the SE strains. The relative expression levels of T1SS and T3SS-1 in the indicated SE strains were determined by qRT-PCR. The mRNA expression levels were normalized against the SE *gyrB* transcript. The parental strain Δ*fliC* was chosen as the calibrator, and the expression levels in other strains were represented relative to that in Δ*fliC*. Data are presented as the mean ± SEM of triplicate samples.
(TIF)

S2 Fig SiiD did not impact the intracellular K^+^ and Ca^2+^ fluxes, the lysosomal rupture, and the expression of NLRP3 inflammasome component proteins. J774A.1 cells were primed with or without LPS (1 μg/mL, 5 h) and then infected with Δ*fliC*, Δ*fliC*Δ*siiD*, Δ*fliC*Δ*siiD*::*siiD*, or Δ*fliC*Δ*siiD*::Vector at an MOI of 100:1 for 4.5 h, uninfected cells were used as a negative control (blank). (A, B) The expression of NLRP3, ASC, pro-Caspase-1, and pro-IL-1β was analyzed by western blotting. β-actin was used as a loading control. Molecular mass markers in kDa are indicated on the right. (C, D, E, F) Cell supernatants and lysates were collected to determine the K^+^ and Ca^2+^ contents. (G) J774A.1 cells primed with LPS and then stimulated with lysosomal rupture inducer LLOMe (1 mM, 2 h) were used as a positive control. Lysosomal rupture was detected by acridine orange (AO) staining. AO fluoresces red in the lysosomes and green in the cytosol. Scale bar: 20 μm. (H) The mean AO red fluorescence was quantified using Application Suite software. Data are presented as the mean ± SEM of triplicate samples per experimental condition from three independent experiments. NS, not significant, as measured by one-way ANOVA followed by Bonferroni’s multiple comparison test.
(TIF)

S3 Fig SiiD inhibited the mtROS dependent NLRP3 inflammasome activation in J774A.1 cells. J774A.1 cells were primed with LPS (1 μg/mL) for 5 h. MitoQ (10 nM) or vehicle control (DMSO) was added to cells 1 h after LPS treatment. The cells were infected with Δ*fliC*, Δ*fliC*Δ*siiD*, Δ*fliC*Δ*siiD*::*siiD*, or Δ*fliC*Δ*siiD*::Vector at an MOI of 100:1 for 4.5 h, uninfected cells were used as a negative control (Blank). (**A**) Cells were then loaded with MitoSOX Red (5 μM) for 30 min. Production of mitochondrial superoxide in infected cells were assayed. Scale bar, 20 μm. (**B**) The mean MitoSOX red fluorescence was quantified using Application Suite software. (**C**) Supernatants were analysed for cytotoxicity evaluated by LDH release. (**D**) The activation of Caspase-1 (p10) and the expression of SiiD were analyzed by western blotting. β-actin was blotted as a loading control. Molecular mass markers in kDa are indicated on the right. (**E**) IL-1β and (**F**) IL-6 secretion in supernatants were examined via ELISA. Data are presented as mean ± SEM of triplicate samples per experimental condition from three independent experiments. **p < 0.01, ***p < 0.001, as measured by one-way ANOVA followed by Bonferroni’s multiple comparison test.
(TIF)

S4 Fig SiiD inhibited the formation of mtROS dependent ASC pyroptosome in J774A.1 cells. J774A.1 cells were primed with LPS (1 μg/mL) for 5 h. MitoQ (10 nM) or vehicle control (DMSO) was added to cells 1 h after LPS treatment. Then cells were infected with Δ*fliC*, Δ*fliC*Δ*siiD*, Δ*fliC*Δ*siiD*::*siiD*, or Δ*fliC*Δ*siiD*::Vector at an MOI of 100:1 for 4.5 h, uninfected cells were used as a negative control (Blank). (**A**) Cells were lysed and the pellets were subjected into cross-link. The ASC oligomerization in the pellets and the total ASC in lysates as the input were examined by western blotting. β-actin was blotted as a loading control. Molecular mass markers in kDa are indicated on the right. (**B**) The formation of ASC specks (arrowheads) was detected by indirect immunofluorescence assay. ASC, red; DAPI, blue. Scale bar, 20 μm. (**C**) The percentages of cells with ASC speck. Approximately 200 cells were counted in each sample. Data are presented as mean ± SEM of triplicate samples per experimental condition from three independent experiments. ***p < 0.001, as measured by one-way ANOVA followed by Bonferroni’s multiple comparison test.
(TIF)

S5 Fig *siiABCDEF* mRNA expression levels in SE collected from infected mice. The WT- and *Nlrp3*^-/—^C57BL/6 mice were orally infected with Δ*fliC* or Δ*fliC*Δ*siiD* at a dose of 5 × 10^6^ CFU per mouse. The spleen, liver, cecum, and cecum contents were harvested and homogenized in sterile PBS at 5 dpi. The relative expression levels of (**A**, **G**) *siiA*, (**B**, **H**) *siiB*, (**C**, **I**) *siiC*, (**D**, **J**) *siiD*, (**E**, **K**) *siiE*, and (**F**, **L**) *siiF* in the SE strains collected from mouse organs were determined by qRT-PCR. The mRNA expression levels were normalized against the SE *gyrB* transcript. The parental strain Δ*fliC* in the logarithmic phase *in vitro* was chosen as the calibrator, and the expression levels in the SE strains *in vivo* and Δ*fliC*Δ*siiD* (in the logarithmic phase *in vitro*) were expressed relative to those in Δ*fliC* (*in vitro*). Data are presented as the mean ± SEM of triplicate samples.
(TIF)
