# Supplementary material for: Salmonella Enteritidis T1SS protein SiiD inhibits NLRP3 inflammasome activation via repressing the mtROS-ASC dependent pathway
Source: PLoS Pathog. 2023 May 8;19(5):e1011381. doi: 10.1371/journal.ppat.1011381 (PMC10194869; doi:10.1371/journal.ppat.1011381)
Supplement: S1 Table — (DOCX) [file ppat.1011381.s001.docx]

**Supporting Information**

**S1 Table.** Bacterial strains and plasmids used in this study

| Strain or plasmid | Relevant characteristics | Reference |
| --- | --- | --- |
| ***Escherichia coli*** |  |  |
| X7213 λ*pir* | Host for π requiring plasmids, conjugal donor | Labortaory collection |
| X7213 λ*pir*-pSC189 | X7213 λ*pir* with pSC189, Km^r^, Cm^r^ | This study |
| DH5α | Δ(*lacZYA-argF*) U169 (Φ80 *LacZ ΔM15*) | Takara |
| BL21 | F^-^, *ompT*, *hsdSB* (rB^-^mB^-^), *gal*, *dcm* | Takara |
| ***Salmonella* Enteritidis** |  |  |
| C50336 | Wild type | Obtained from Chinese National Institute for the Control of Pharmaceutical and Biological |
| ∆*fliC* | C50336, In-frame deletion in *fliC* | Labortaory collection |
| ∆*fliC*∆*siiD* | C50336, In-frame deletion in *fliC* and *siiD* | This study |
| ∆*fliC*∆*rcsD* | C50336, In-frame deletion in *fliC* and *rcsD* | This study |
| ∆*fliC*∆*siiC* | C50336, In-frame deletion in *fliC* and *siiC* | This study |
| ∆*fliC*∆*sifA* | C50336, In-frame deletion in *fliC* and *sifA* | This study |
| ∆*fliC*∆*siiD*::*siiD* | ∆*fliC*∆*siiD* with pBAD33 expressing the *siiD* gene, Cm^r^ | This study |
| ∆*fliC*∆*siiD*::Vector | ∆*fliC*∆*siiD* with pBAD33, Cm^r^ | This study |
| ∆*fliC*∆*prgH* | C50336, In-frame deletion in *fliC* and *prgH* | This study |
| ∆*fliC*∆*ssaV* | C50336, In-frame deletion in *fliC* and *ssaV* | This study |
| ∆*fliC*∆T1SS | C50336, In-frame deletion in *fliC* and *SPI-4* | This study |
| ∆*fliC*-pCX340 | ∆*fliC* with pCX340, Tet^r^ | This study |
| ∆*fliC*∆*siiD*-pCX340-*siiD* | ∆*fliC*∆*siiD* with pCX340 expressing the *siiD* gene, Tet^r^ | This study |
| ∆*fliC*∆T3SS-1-pCX340-*siiD* | ∆*fliC*∆*prgH* with pCX340 expressing the *siiD* gene, Tet^r^ | This study |
| ∆*fliC*∆T3SS-2-pCX340-*siiD* | ∆*fliC*∆*ssaV* with pCX340 expressing the *siiD* gene, Tet^r^ | This study |
| ∆*fliC*∆T1SS-pCX340-*siiD* | ∆*fliC*∆T1SS with pCX340 expressing the *siiD* gene, Tet^r^ | This study |
| ∆*fliC*∆*siiD*::*siiD*-HA | ∆*fliC*∆*siiD* with pBAD33 expressing the *siiD*-HA, Cm^r^ | This study |
| ∆*fliC*∆T3SS-1::*siiD*-HA | ∆*fliC*∆*prgH* with pBAD33 expressing the *siiD*-HA, Cm^r^ | This study |
| ∆*fliC*∆T3SS-2::*siiD*-HA | ∆*fliC*∆*ssaV* with pBAD33 expressing the *siiD*-HA, Cm^r^ | This study |
| ∆*fliC*∆T1SS::*siiD*-HA | ∆*fliC*∆T1SS with pBAD33 expressing the *siiD*-HA, Cm^r^ | This study |
| **Plasmids** |  |  |
| pSC189 | Transposon delivery vector, R6K, Km^r^, Cm^r^ | [1] |
| pDM4 | Suicide vector, *pir* dependent, R6K, *SacBR*, Cm^r^ | [2] |
| pBAD33 | p15A-based expression vector utilizing P_BAD_ promoter, Cm^r^ | [3] |
| pBAD33-*siiD* | pBAD33 derivative containing *siiD*, Cm^r^ | This study |
| pBAD33-*siiD*-HA | pBAD33 derivative containing *siiD*-HA, Cm^r^ | This study |
| pGEX-6p-1 | lacI, tac promoter and glutathione S-transferase (GST) , Amp^r^ | Labortaory collection |
| pGEX-6p-1-*siiD* | pGEX-6p-1 derivative containing *siiD*, Amp^r^ | This study |
| pCX340 | pBR322 derivative, cloning vector used to fuse effectors to TEM-1-β-lactamase, Tet^r^ | [4] |
| pCX340-*siiD* | pCX340 derivative containing *siiD* | This study |

The antibiotics as follows: kanamycin (Km^r^), 100 μg/mL; chloramphenicol (Cm^r^), 25 μg/mL; tetracycline (Tet^r^), 12.5 μg/mL.

**References**

1. Chiang SL, Rubin EJ. Construction of a mariner-based transposon for epitope-tagging and genomic targeting. Gene. 2002;296(1-2):179-85. Epub 2002/10/18. doi: 10.1016/s0378-1119(02)00856-9. PubMed PMID: 12383515.

2. Wang SY, Lauritz J, Jass J, Milton DL. A ToxR homolog from Vibrio anguillarum serotype O1 regulates its own production, bile resistance, and biofilm formation. J Bacteriol. 2002;184(6):1630-9. Epub 2002/03/02. doi: 10.1128/JB.184.6.1630-1639.2002. PubMed PMID: 11872714; PubMed Central PMCID: PMCPMC134897.

3. Guzman LM, Belin D, Carson MJ, Beckwith J. Tight regulation, modulation, and high-level expression by vectors containing the arabinose PBAD promoter. J Bacteriol. 1995;177(14):4121-30. Epub 1995/07/01. doi: 10.1128/jb.177.14.4121-4130.1995. PubMed PMID: 7608087; PubMed Central PMCID: PMCPMC177145.

4. Charpentier X, Oswald E. Identification of the secretion and translocation domain of the enteropathogenic and enterohemorrhagic Escherichia coli effector Cif, using TEM-1 beta-lactamase as a new fluorescence-based reporter. J Bacteriol. 2004;186(16):5486-95. Epub 2004/08/05. doi: 10.1128/JB.186.16.5486-5495.2004. PubMed PMID: 15292151; PubMed Central PMCID: PMCPMC490934.
