# Supplementary material for: Salmonella Enteritidis T1SS protein SiiD inhibits NLRP3 inflammasome activation via repressing the mtROS-ASC dependent pathway
Source: PLoS Pathog. 2023 May 8;19(5):e1011381. doi: 10.1371/journal.ppat.1011381 (PMC10194869; doi:10.1371/journal.ppat.1011381)
Supplement: S2 Table — (DOCX) [file ppat.1011381.s002.docx]

**Supporting Information**

**S2 Table.** Primers used in this study

| Primer name | Primer sequence (5’ to 3’) | Target |
| --- | --- | --- |
| Km-out-F | AGGATCGTTTCGCATGATTG | For transposon mutants |
| Km-out-R | GTCCCGCTCAGAAGAACTCG |  |
| AB1 | GGCCACGCGTCGACTAGTACNNNNNNNNNNACGCC | For transposon insertion sequencing |
| AB2 | GGCCACGCGTCGACTAGTACNNNNNNNNNNCCTGG |  |
| AB3 | GGCCACGCGTCGACTAGTACNNNNNNNNNNCCTCG |  |
| ABS | GGCCACGCGTCGACTAGTAC |  |
| SP1 | GCTGACCGCTTCCTCGTGCTTTACG |  |
| SP2 | CATCGCCTTCTATCGCCTTCTTGAC |  |
| pSC189-seq | CGCGAAGTTCCTATTCCGAAGTTCC |  |
| pDM4-F | GGTGCTCCAGTGGCTTCTGTTTCTA | For deletion mutants |
| pDM4-R | CAGCAACTTAAATAGCCTCTAAT |  |
| *siiD*-up-F | GAGCGGATAACAATTTGTGGAATCCCGGGACGCAAAATCATCCTATTTTCCGACT | For *siiD* deletion mutant |
| *siiD*-up-R | TTCACTCTCATCATTTCATTACATTTAACTCACTT |  |
| *siiD*-down-F | AATGAAATGATGAGAGTGAATATAATATTATGGGA |  |
| *siiD*-down-R | AGCGGAGTGTATATCAAGCTTATCGATACCCATCGCTTTGCTGAGCCAGATTGTC |  |
| *siiD*-in-F | GATTACCCTGGATGGCACCCGTTCT |  |
| *siiD*-in-R | CTGAATTGTTCCTCCGGTATTTTCC |  |
| *siiD*-out-F | TTTATCGCCAGTTCAGATTCAAAGC |  |
| *siiD*-out-R | GAAGCCTGCGTTGCTTTAGCCTGTG |  |
| *rcsD*-up-F | GAGCGGATAACAATTTGTGGAATCCCGGGACAAGTAAAAATAAGCACAAATAGAC | For *rcsD* deletion mutant |
| *rcsD*-up-R | TACCTTGCTACATTTATGTTTACGACCTGTAAAAG |  |
| *rcsD*-down-F | AACATAAATGTAGCAAGGTAGCCCAATACATGAAC |  |
| *rcsD*-down-R | AGCGGAGTGTATATCAAGCTTATCGATACCGATTTCTTCTGGCTGCTGATGGTCT |  |
| *rcsD*-in-F | ACGACCTTCAATCAGCCAGGACATC |  |
| *rcsD-*in-R | AAGCCCGAGGCAGTAAGATTAGACG |  |
| *rcsD*-out-F | TCCAACACGAATATGATACTAAAAC |  |
| *rcsD*-out-R | AAGTGATTTATTCTTTGTCTGTCGG |  |
| *siiC*-up-F | GAGCGGATAACAATTTGTGGAATCCCGGGATGGATAACTCAGCAAAACTGGAACA | For *siiC* deletion mutant |
| *siiC*-up-R | TTCTATTCATTTAATCTTCATTTTTTTCCTCCTTG |  |
| *siiC*-down-F | TGAAGATTAAATGAATAGAAGACAAAGCGATCATC |  |
| *siiC*-down-R | AGCGGAGTGTATATCAAGCTTATCGATACCTTTTTTTACCAGTGGGGAAAGAATA |  |
| *siiC*-in-F | CTGACGACTGCTTTTATTACACAAA |  |
| *siiC-*in-R | CAGTATTTCCTTTTAAATAATCCAT |  |
| *siiC*-out-F | GGACGCAGGATGAAGAAAAGAAACG |  |
| *siiC*-out-R | ATATCCTCCTCATTTATCTGCTTCT |  |
| *sifA*-up-F | GAGCGGATAACAATTTGTGGAATCCCGGGAGGCAATGGGCCTGTTCTACGTCTCC | For *sifA* deletion mutant |
| *sifA*-up-R | TCTGATTTTACATATTAATCTCACTTAAACTGGAG |  |
| *sifA*-down-F | GATTAATATGTAAAATCAGACGACGCTTTCTCAAA |  |
| *sifA*-down-R | AGCGGAGTGTATATCAAGCTTATCGATACCCGAGCCAATCAACGTGGCGAAGGTC |  |
| *sifA*-in-F | AAATGATGCCACCATTATTCTTCGC |  |
| *sifA-*in-R | GGTATGTGGGTATGCGGTGGTGGTA |  |
| *sifA*-out-F | CTGGGCGTGATTGATACACCGATTC |  |
| *sifA*-out-R | TGACGACCACAAACGGCAGGCTAAA |  |
| *prgH*-up-F | GAGCGGATAACAATTTGTGGAATCCCGGGACACCAACATCCCAGGTTCGTCACAG | For *prgH* deletion mutant |
| *prgH*-up-R | CGTTAAATTACATATATACTGTTAGCGATGTCTGT |  |
| *prgH*-down-F | AGTATATATGTAATTTAACGTAAATAAGGAAGTCA |  |
| *prgH*-down-R | AGCGGAGTGTATATCAAGCTTATCGATACCTTCAACAGCCCCGACTCCTTTACGA |  |
| *prgH*-in-F | GTTTGCTGCTCGTTTGGGATAAGTG |  |
| *prgH*-in-R | GGCAAGGGTCATTACCAGCAGAAAG |  |
| *prgH*-out-F | GAACGGCTGTGAGTTTCCATTGCTG |  |
| *prgH*-out-R | GACGGGCTCTGAGTATTTCTACATC |  |
| *ssaV*-up-F | GAGCGGATAACAATTTGTGGAATCCCGGGAATCGAAGATGTTCAATCGGTTACTC | For *ssaV* deletion mutant |
| *ssaV*-up-R | CATTCTTCATCTAACCATGAACGCATTGCGACTCC |  |
| *ssaV*-down-F | TCATGGTTAGATGAAGAATGAATTGATGCAACGTC |  |
| *ssaV*-down-R | AGCGGAGTGTATATCAAGCTTATCGATACCCACCAGAACATTGCTGTCTGCGTCT |  |
| *ssaV*-in-F | AACAGTGGCTCAGTGTATGCGCGGG |  |
| *ssaV-*in-R | AATAGAAACCCGTTCTGATACAAGC |  |
| *ssaV*-out-F | GATTTGCCAGTTGCCGCCTAATACA |  |
| *ssaV*-out-R | GTCTGTCAGAGGTTGCGACAACAAT |  |
| *Spi-4*-up-F | GAGCGGATAACAATTTGTGGAATCCCGGGAATTCTGATTTTTTACTCCCCACTTA | For *Spi-4* deletion mutant |
| *Spi-4*-up-R | GCTTATTTTACATGTTGTCTCCTGATATTACATTG |  |
| *Spi-4*-down-F | AGACAACATGTAAAATAAGCAGCGCTTGTCGCTGT |  |
| *Spi-4*-down-R | AGCGGAGTGTATATCAAGCTTATCGATACCCTGGACAAAGGTCTATTCAGAGGAA |  |
| *Spi-4*-in-F | CTCGATCTTTCAACGTATAGCCGAT |  |
| *Spi-4*-in-R | TCACTACTCAGACTACGTGCTAATA |  |
| *Spi-4*-out-F | ACTCGCCTCGGCACATCATTATCCA |  |
| *Spi-4*-out-R | TCCGTTGATAGCCAGAATGTCCCAC |  |
| pBAD33-F | TCTACTGTTTCTCCATACCCGTTTT | For complemented mutants |
| pBAD33-R | TTCTGCGTTCTGATTTAATCTGTAT |  |
| pBAD33-*siiD*-F | GGGCTAGCGAATTCGAGCTCGGTACCTAAAGGAAGACGTTATGAATAGAAGACAAAGCGATCATC | For complemented mutant of ∆*siiD* |
| pBAD33-*siiD*-HA-R | TCTCATCCGCCAAAACAGCCAAGCTTCAAGCGTAATCTGGAACATCGTATGGGTAAGGTGTATCTAATCGTTTAGTA |  |
| pCX340-F | AGACAATCTGTGTGGGCACTCGACC | For β-lactamase TEM-1 fusion plasmid |
| pCX340-R | TTCTGAGAATAGTGTATGCGGCGAC |  |
| pCX340-*siiD*-F | AATAAGGAGGAATAACATATGATGAATAGAAGACAAAGCGATCATC |  |
| pCX340-*siiD*-R | CGAATTCTCCGCGGAGGTACCAGGTGTATCTAATCGTTTAGTA |  |
| pBAD33-*siiD*-F | GGGCTAGCGAATTCGAGCTCGGTACCTAAAGGAAGACGTTATGAATAGAAGACAAAGCGATCATC | For complemented mutant of ∆*siiD* |
| pBAD33-*siiD*-R | TCTCATCCGCCAAAACAGCCAAGCTTCAAGGTGTATCTAATCGTTTAGTA |  |
| pGEX-6p-1-*siiD*-F | TCTGTTCCAGGGGCCCCTGGGATCCATGAATAGAAGACAAAGCGATCATC | For prokaryotic expression of SiiD |
| pGEX-6p-1-*siiD*-R | GTCAGTCACGATGCGGCCGCTCGAGAGGTGTATCTAATCGTTTAGTA |  |
| pGEX-6p-1-F | GGGCTGGCAAGCCACGTTTGGTG | For prokaryotic expression of SiiD |
| pGEX-6p-1-R | CCGGGAGCTGCATGTGTCAGAGG |  |
