# Supplementary material for: Salmonella Enteritidis T1SS protein SiiD inhibits NLRP3 inflammasome activation via repressing the mtROS-ASC dependent pathway
Source: PLoS Pathog. 2023 May 8;19(5):e1011381. doi: 10.1371/journal.ppat.1011381 (PMC10194869; doi:10.1371/journal.ppat.1011381)
Supplement: S3 Table — (DOCX) [file ppat.1011381.s003.docx]

**Supporting Information**

**S3 Table.** Primers used for qRT-PCR in this study

| Primer name | Primer sequence (5’ to 3’) |
| --- | --- |
| *siiA*-RT-F | CGAAAGTAATCCGTGGCCTA |
| *siiA*-RT-R | GCTCCGCTATTTTGAGCAGT |
| *siiB*-RT-F | CGTTTGCGGGTCTGTCTATT |
| *siiB*-RT-R | GCGGTATTCATCCCTTTCAA |
| *siiC*-RT-F | TGCCGCAAAATCATCCTATT |
| *siiC*-RT-R | GCGAATAACTTGCCACCATT |
| *siiD*-RT-F | CGGCGGACTTACTTTTTGAA |
| *siiD*-RT-R | GCTGAATTGTTCCTCCGGTA |
| *siiE*-RT-F | CAATGAAGTCCGTCTGAGCA |
| *siiE*-RT-R | CGCGCTATTCAGTACGATGA |
| *siiF*-RT-F | GCTACCTTTCGCCAACAGAC |
| *siiF*-RT-R | GCGGTTTTAACGCGAAATTA |
| *spaP*-RT-F | ATCGCGATAACCATCCAGAC |
| *spaP*-RT-R | CGTCAGGAACCTGTTTCGTT |
| *spaQ*-RT-F | TAGCCAACGCCAGGAATATC |
| *spaQ*-RT-R | GACGATTGTCGCAACGATTA |
| *spaR*-RT-F | CAGACCGCCGTTTTGTAAAT |
| *spaR*-RT-R | CATGGCCTTTTTGGGTTATG |
| *spaS*-RT-F | GCCTGTAATAACGCCGGTAA |
| *spaS*-RT-R | TGGGAGGAATTGCCTATCTG |
| *prgH*-RT-F | GCTGCGGCGAGTTAAGTATC |
| *prgH*-RT-R | GCCAATACAGGTCGGTGAAT |
| *prgI*-RT-F | ATTGCGCGTTACGGTACAAG |
| *prgI*-RT-R | CTGGATGACGTCTCAGCAAA |
| *prgJ*-RT-F | AACAGCCCCGACTCCTTTAC |
| *prgJ*-RT-R | GACATTGTCTCGCTGGATGA |
| *prgK*-RT-F | TCATAATCCACATCGGCAAA |
| *prgK*-RT-R | TTGAACAGCGACTGGAACAG |
| *invA*-RT-F | CTCGCCTTTGCTGGTTTTAG |
| *invA*-RT-R | GTGCCGGTTTTATCGTGACT |
| *invG*-RT-F | CGTAAAGAAACCACGGCATT |
| *invG*-RT-R | ATGGCGCTACAGCTAAAGGA |
| *sipB*-RT-F | TCGCAGCGTCATAAACACTC |
| *sipB*-RT-R | TGTTTCGCTGTCTCAACTGG |
| *sipC*-RT-F | AACGGCACTGGAAGACATTC |
| *sipC*-RT-R | AAGTCAGTGACCTGGGGTTG |
| *sipD*-RT-F | GCGCTGGAAATAAAACGGTA |
| *sipD*-RT-R | TGGGATATGGTTTCCCAAAA |
| *gyrB*-RT-F | TGATTGCGGTGGTTTCCGTA |
| *gyrB*-RT-R | GACGACGATTTTCGCGTCAG |
