# Supplementary figures and images for: Salmonella Enteritidis T1SS protein SiiD inhibits NLRP3 inflammasome activation via repressing the mtROS-ASC dependent pathway
Source: PLoS Pathog. 2023 May 8;19(5):e1011381. doi: 10.1371/journal.ppat.1011381 (PMC10194869; doi:10.1371/journal.ppat.1011381)

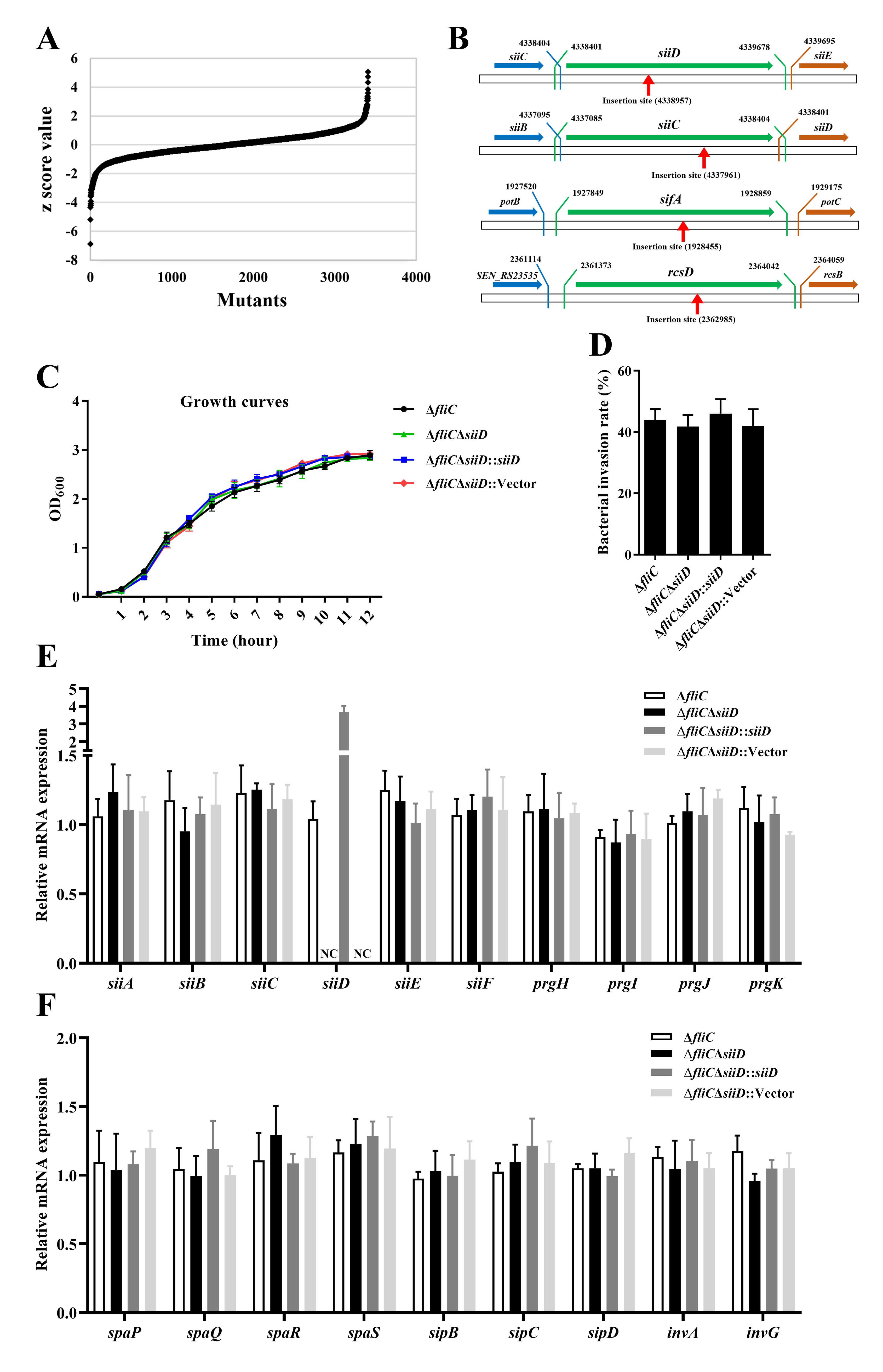

Supplement: S1 Fig — (A) Distribution of Z score data of cytotoxicity levels induced by 3409 ΔfliC transposon mutants. The Z score was calculated for each well in a 48-well cell plate, and a Z score ≤ -2 or ≥ 2 was considered significant. (B) The transposon insertion sites of each candidate transposon mutants. Horizontal arrows indicate the direction of gene expression. Candidate gene, green; Upstream gene, blue; Downstream gene, brown. The numbers represent the initial or terminal position in the SE genome of each gene. The red vertical arrows represent the transposon insertion site of each candidate transposon mutants. (C) Growth curves of ΔfliC, ΔfliCΔsiiD, ΔfliCΔsiiD::siiD, and ΔfliCΔsiiD::Vector. Bacteria were grown in liquid LB medium at 37°C for 12 h with agitation, and the OD600 values of triplicate cultures in LB medium were determined in 1-h intervals. (D) J774A.1 cells were infected with ΔfliC, ΔfliCΔsiiD, ΔfliCΔsiiD::siiD, or ΔfliCΔsiiD::Vector at an MOI of 100:1 for 1.5 h. Bacterial lysates were plated to determine the invasiveness of SE strains. (E, F) J774A.1 cells were infected with the indicated SE strains at an MOI of 100:1 for 4.5 h. Bacterial lysates were plated to determine the invasiveness of the SE strains. The relative expression levels of T1SS and T3SS-1 in the indicated SE strains were determined by qRT-PCR. The mRNA expression levels were normalized against the SE gyrB transcript. The parental strain ΔfliC was chosen as the calibrator, and the expression levels in other strains were represented relative to that in ΔfliC. Data are presented as the mean ± SEM of triplicate samples. (TIF) [file ppat.1011381.s004.tif]

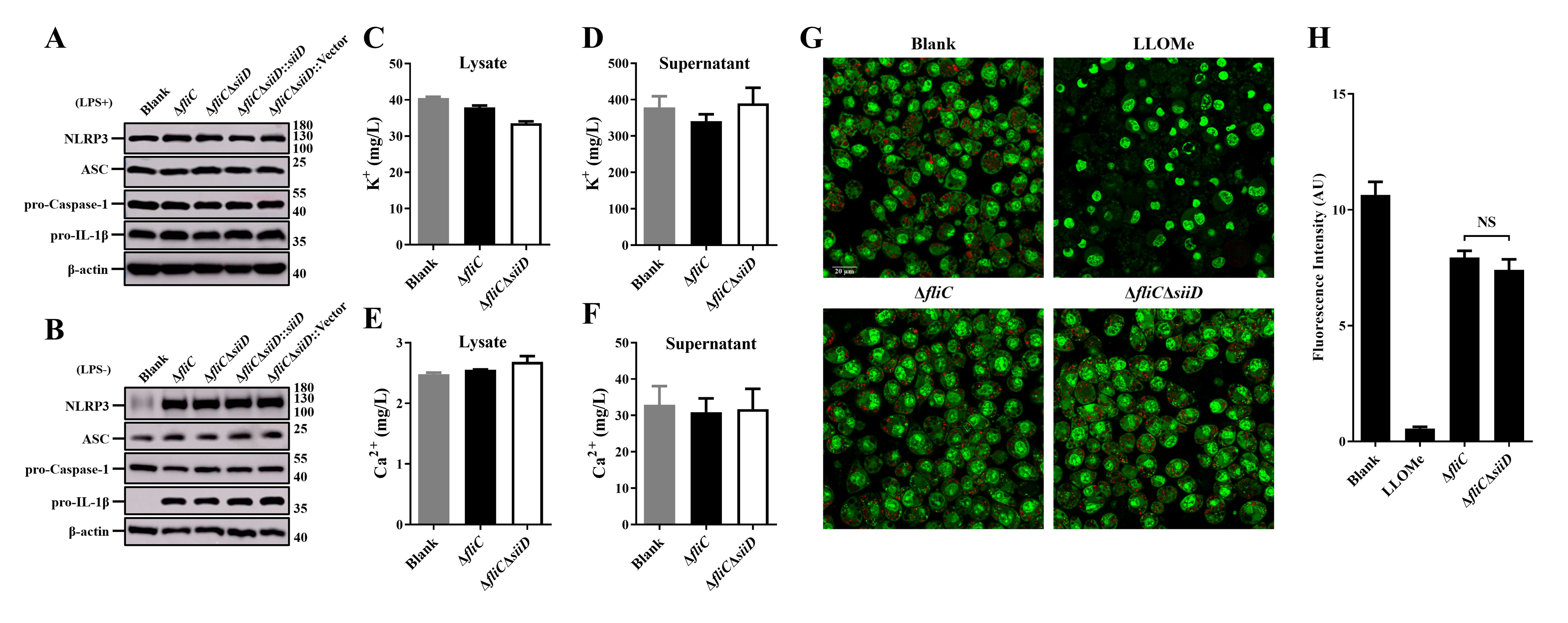

Supplement: S2 Fig — J774A.1 cells were primed with or without LPS (1 μg/mL, 5 h) and then infected with ΔfliC, ΔfliCΔsiiD, ΔfliCΔsiiD::siiD, or ΔfliCΔsiiD::Vector at an MOI of 100:1 for 4.5 h, uninfected cells were used as a negative control (blank). (A, B) The expression of NLRP3, ASC, pro-Caspase-1, and pro-IL-1β was analyzed by western blotting. β-actin was used as a loading control. Molecular mass markers in kDa are indicated on the right. (C, D, E, F) Cell supernatants and lysates were collected to determine the K+ and Ca2+ contents. (G) J774A.1 cells primed with LPS and then stimulated with lysosomal rupture inducer LLOMe (1 mM, 2 h) were used as a positive control. Lysosomal rupture was detected by acridine orange (AO) staining. AO fluoresces red in the lysosomes and green in the cytosol. Scale bar: 20 μm. (H) The mean AO red fluorescence was quantified using Application Suite software. Data are presented as the mean ± SEM of triplicate samples per experimental condition from three independent experiments. NS, not significant, as measured by one-way ANOVA followed by Bonferroni’s multiple comparison test. (TIF) [file ppat.1011381.s005.tif]

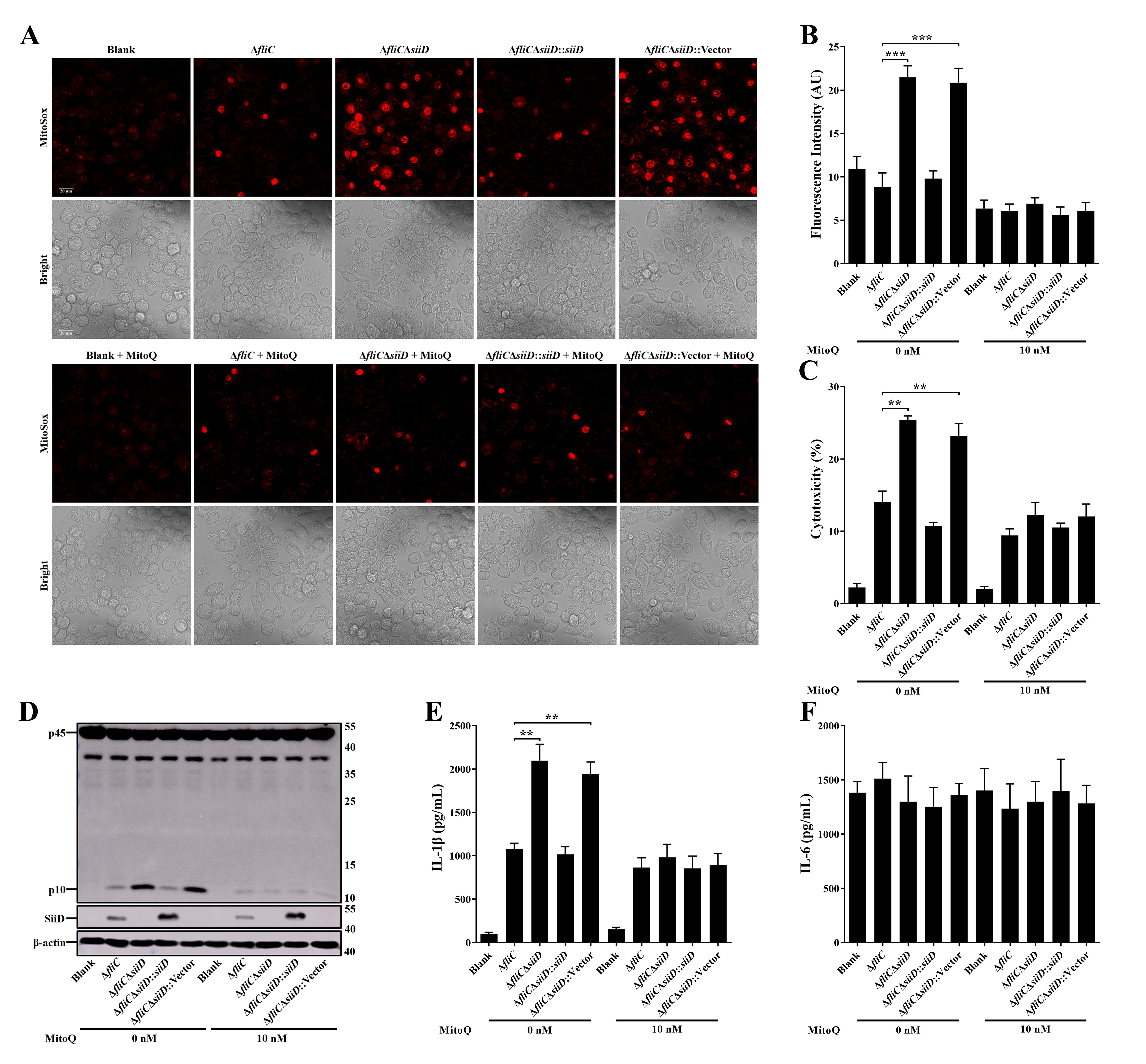

Supplement: S3 Fig — J774A.1 cells were primed with LPS (1 μg/mL) for 5 h. MitoQ (10 nM) or vehicle control (DMSO) was added to cells 1 h after LPS treatment. The cells were infected with ΔfliC, ΔfliCΔsiiD, ΔfliCΔsiiD::siiD, or ΔfliCΔsiiD::Vector at an MOI of 100:1 for 4.5 h, uninfected cells were used as a negative control (Blank). (A) Cells were then loaded with MitoSOX Red (5 μM) for 30 min. Production of mitochondrial superoxide in infected cells were assayed. Scale bar, 20 μm. (B) The mean MitoSOX red fluorescence was quantified using Application Suite software. (C) Supernatants were analysed for cytotoxicity evaluated by LDH release. (D) The activation of Caspase-1 (p10) and the expression of SiiD were analyzed by western blotting. β-actin was blotted as a loading control. Molecular mass markers in kDa are indicated on the right. (E) IL-1β and (F) IL-6 secretion in supernatants were examined via ELISA. Data are presented as mean ± SEM of triplicate samples per experimental condition from three independent experiments. **p < 0.01, ***p < 0.001, as measured by one-way ANOVA followed by Bonferroni’s multiple comparison test. (TIF) [file ppat.1011381.s006.tif]

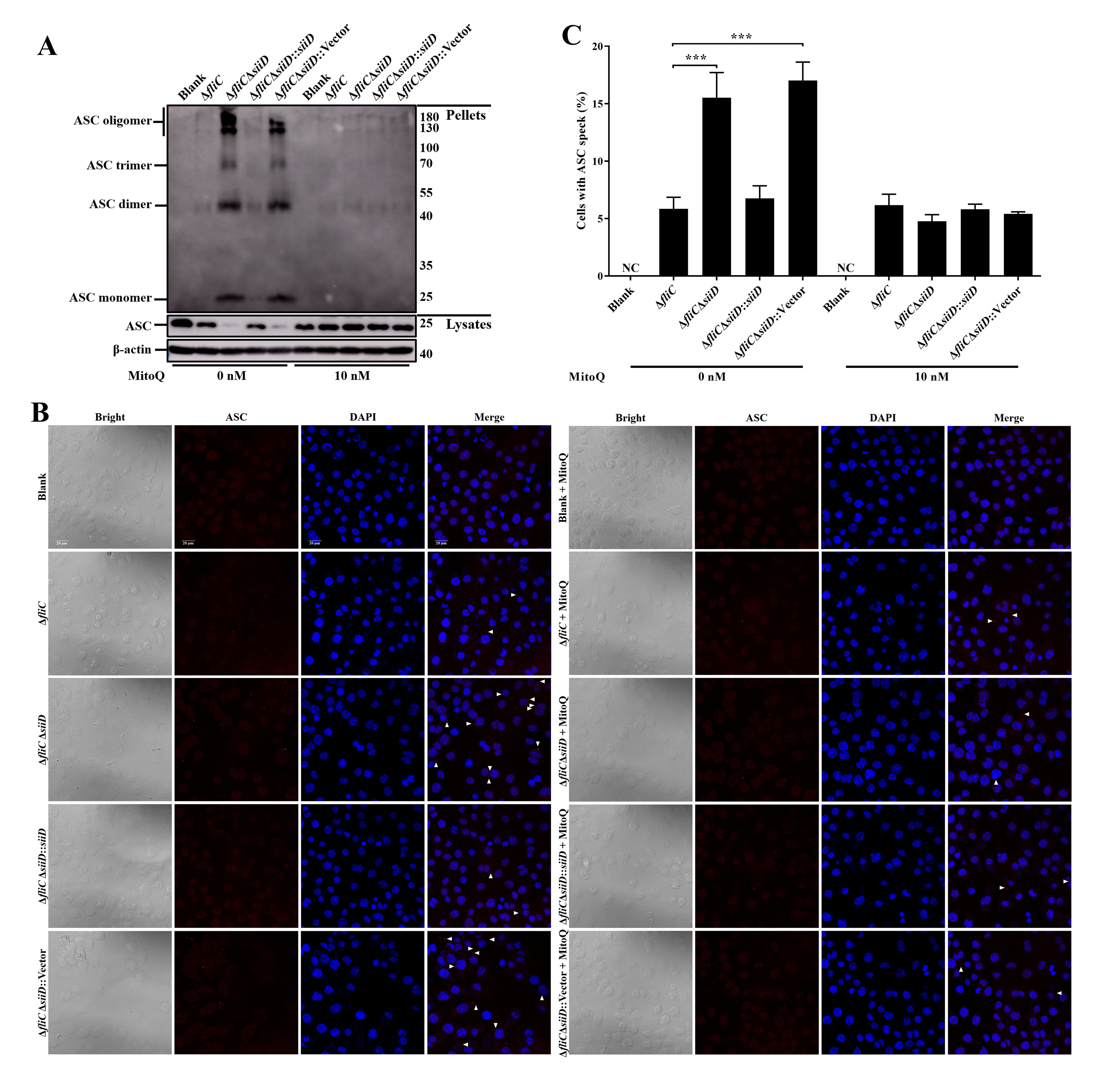

Supplement: S4 Fig — J774A.1 cells were primed with LPS (1 μg/mL) for 5 h. MitoQ (10 nM) or vehicle control (DMSO) was added to cells 1 h after LPS treatment. Then cells were infected with ΔfliC, ΔfliCΔsiiD, ΔfliCΔsiiD::siiD, or ΔfliCΔsiiD::Vector at an MOI of 100:1 for 4.5 h, uninfected cells were used as a negative control (Blank). (A) Cells were lysed and the pellets were subjected into cross-link. The ASC oligomerization in the pellets and the total ASC in lysates as the input were examined by western blotting. β-actin was blotted as a loading control. Molecular mass markers in kDa are indicated on the right. (B) The formation of ASC specks (arrowheads) was detected by indirect immunofluorescence assay. ASC, red; DAPI, blue. Scale bar, 20 μm. (C) The percentages of cells with ASC speck. Approximately 200 cells were counted in each sample. Data are presented as mean ± SEM of triplicate samples per experimental condition from three independent experiments. ***p < 0.001, as measured by one-way ANOVA followed by Bonferroni’s multiple comparison test. (TIF) [file ppat.1011381.s007.tif]

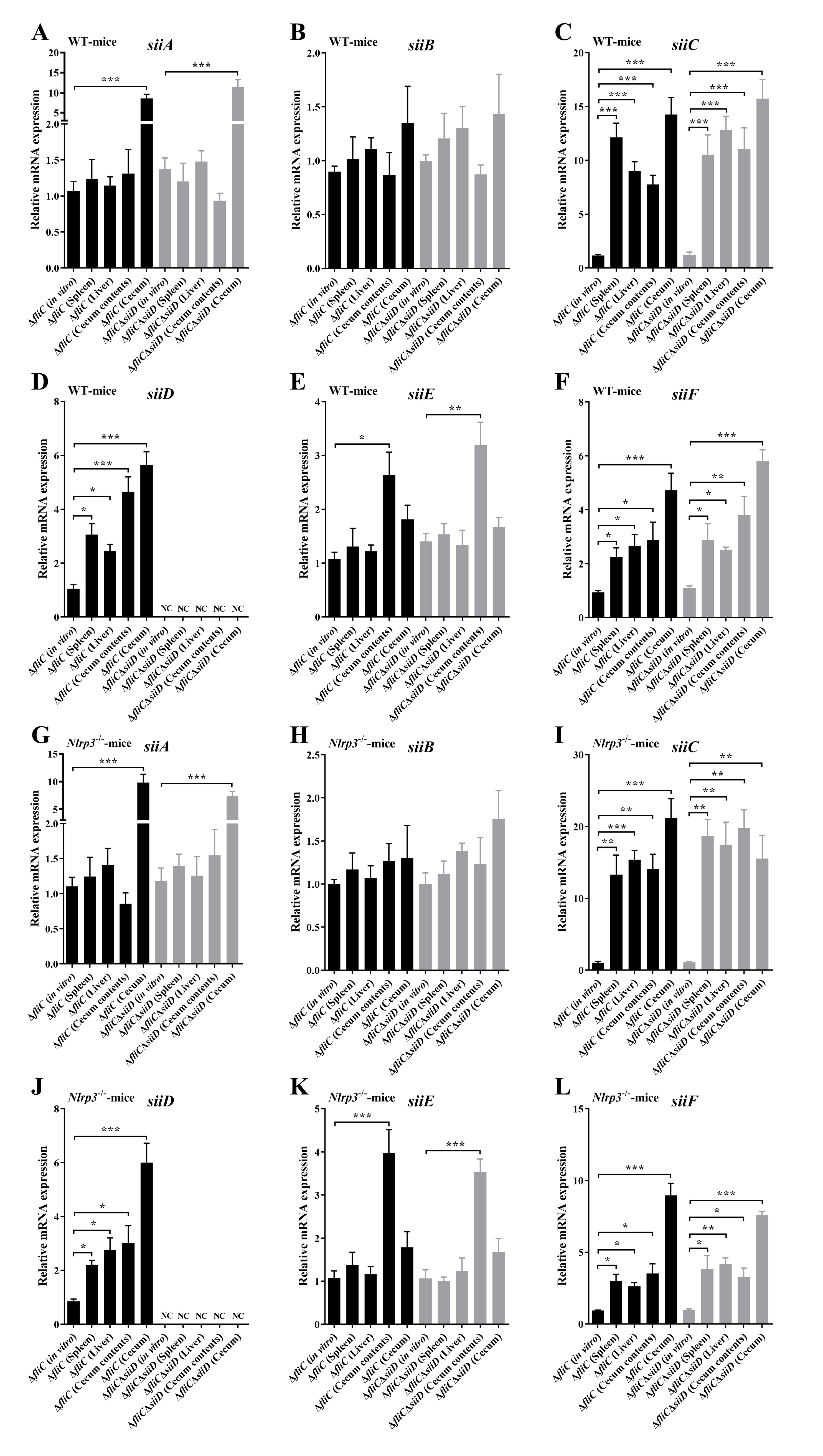

Supplement: S5 Fig — The WT- and Nlrp3-/—C57BL/6 mice were orally infected with ΔfliC or ΔfliCΔsiiD at a dose of 5 × 10^6^ CFU per mouse. The spleen, liver, cecum, and cecum contents were harvested and homogenized in sterile PBS at 5 dpi. The relative expression levels of (A, G) siiA, (B, H) siiB, (C, I) siiC, (D, J) siiD, (E, K) siiE, and (F, L) siiF in the SE strains collected from mouse organs were determined by qRT-PCR. The mRNA expression levels were normalized against the SE gyrB transcript. The parental strain ΔfliC in the logarithmic phase in vitro was chosen as the calibrator, and the expression levels in the SE strains in vivo and ΔfliCΔsiiD (in the logarithmic phase in vitro) were expressed relative to those in ΔfliC (in vitro). Data are presented as the mean ± SEM of triplicate samples. (TIF) [file ppat.1011381.s008.tif]
